# A thermochronological transect across the Trento platform: constraints for the evolution of the European Eastern Southern Alps

**DOI:** 10.1186/s00015-025-00491-w

**Published:** 2025-09-25

**Authors:** Thomas Klotz, Anna-Katharina Sieberer, István Dunkl, Paul R. Eizenhöfer, Hannah Pomella

**Affiliations:** 1 Department of Geology, University of Innsbruck, 6020 Innsbruck, Austria; 2 Department of Sedimentology and Environmental Geology, University of Göttingen, 37077 Göttingen, Germany; 3 School of Geographical & Earth Sciences, University of Glasgow, Glasgow, G12 8QQ Scotland

**Keywords:** Thermochronology, Tectonics, Fold-and-thrust belt evolution, Exhumation, Eastern Southern Alps, Dolomites, Dolomites Indenter, Neoalpine, Dinaric

## Abstract

Late Oligocene and Miocene east–west decoupling of the Alpine retro-wedge evolution along the Giudicarie fault system and the associated late-stage shortening within the eastern Southern Alps (ESA) are key features of the Neoalpine, post-collisional Adria-Europe convergence. A new thermochronological N-S transect across the Trento platform in the western ESA suggests a ~ 25 Ma onset of exhumation in the Neoalpine fold-and-thrust belt. This requires a longer lasting transfer of shortening from the Eastern Alps, north of the Periadriatic fault system, into the evolving ESA, rather than a single and distinct Middle Miocene shift to a coupled state. Still, the highest exhumation rates are observed between 17 and 10 Ma, linking rapid ESA exhumation to the termination of folding within the Sub-Penninic interior of the Tauern Window. Prior to the Neoalpine evolution, the oblique Paleogene collision of northeast-Adria and Europe entailed Dinaric top-southwest thrusting on the Adriatic microplate. An Eocene antiformal basement structure and an assumed blind fault in the northwestern ESA, revealed by new thermochronological data, mark the northwestern extend of the Dinaric fold-and-thrust belt. Apart from Dinaric and Neoalpine exhumation related cooling, Adria was affected by Eocene to Oligocene subduction-related magmatism, Late Triassic–Early Jurassic extension, middle Triassic strike-slip tectonics, and Permian extension. Each event, as well as the Permian to Cretaceous burial, resulted in the thermal perturbation of the Adriatic crust and its sedimentary cover. Statistical analyses of apatite fission-track single-grain ages emphasize the imprint of the post-Permian thermal evolution, as detrital ages and magmatic formation ages are overprinted or obliterated throughout the study area. Jurassic and Cretaceous apatite fission-track data are attributed to a widespread exhumed partial annealing zone.

**Supplementary Information:**

The online version contains supplementary material available at 10.1186/s00015-025-00491-w.

## Introduction

**Fig. 1:**
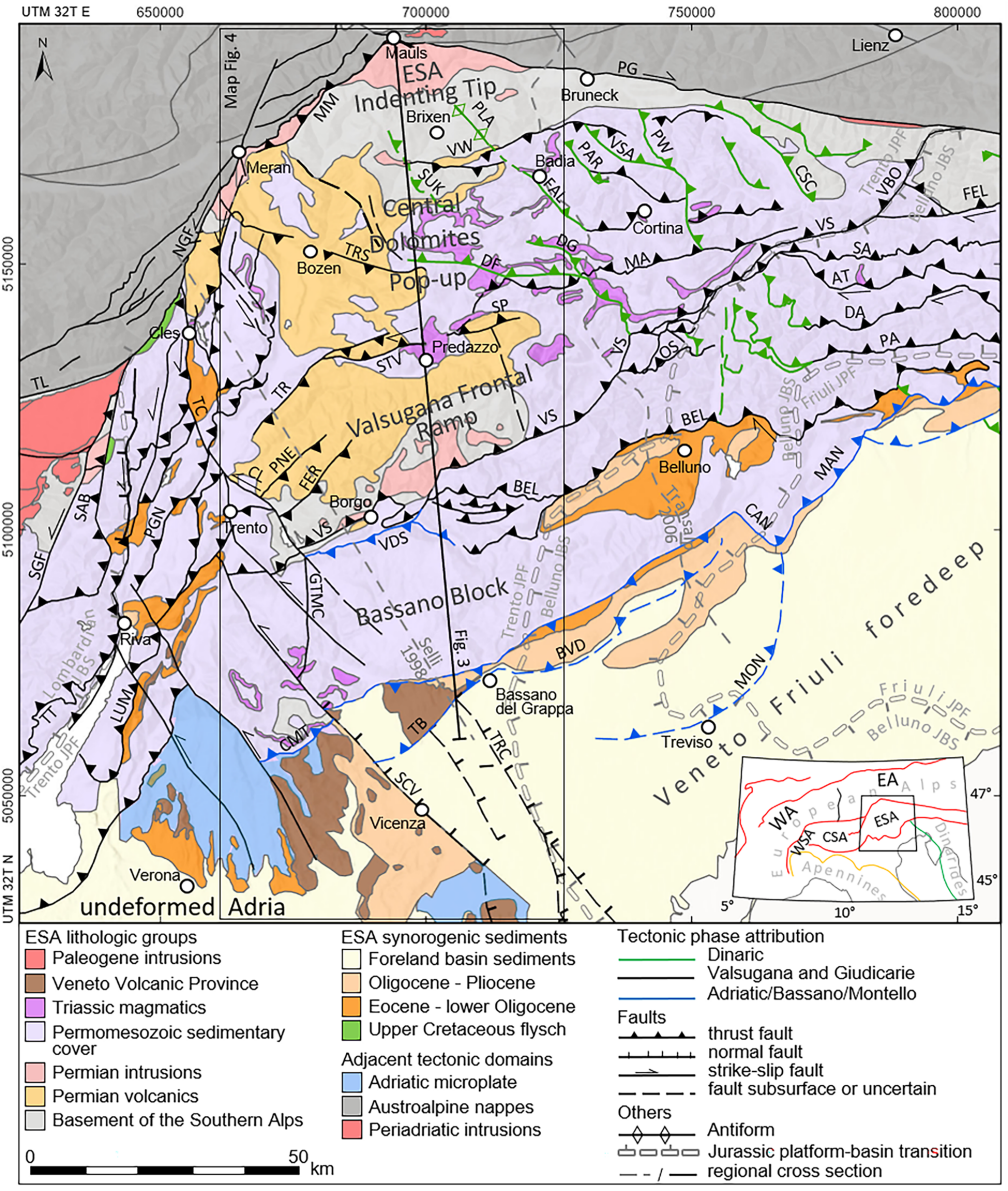
Geological map of the eastern Southern Alps. *AT* Ampezzo-Tolmezzo thrust, *BEL* Belluno thrust, *BVD* Bassano-Valdobbiadene thrust, *CAN* Cansiglio fault, *CL* Calisio fault, *CMT* Cima Marana thrust, *CSA* central Southern Alps, *CSC* Candido-S. Stefano di Cadore line, *DA* Dof-Auda thrust, *DF* Duron-Fedaia line, *DG* Digonera thrust, *EA* Eastern Alps, *ESA* eastern Southern Alps, *FAL* Falzarego line, *FEL* Fella fault, *GTMC* Gamonda/Tormeno/Melegnon/Carotte fault, *JPF* Jurassic platform, *JBS* Jurassic basin, *LUM* Lumini fault, *MA* Marmolada-Antelao thrust, *MAN* Maniago thrust, *MM* Meran-Mauls fault, *MON* Montello thrust, *NGF* Northern Giudicarie fault, *OS* Ospitale line, *PA* Pinedo-Avasinis thrust, *PAC* Plose anticline, *PAR* Monte Parei-Col Becchei-Fanes thrust, *PG* Pustertal-Gailtal fault, *PGN* Paranella fault, *PLA* Plose Antiform, *PNE* Pine line, *PW* Plätzwiese line, *SA* Sauris thrust, *SAB* Sabion line, *SCV* Schio-Vicenza fault, *SGF* Southern Giudicarie fault, *SP* San Pellegrino line, *STV* Stava line, *SUK* St. Ulrich-Klausen fault, *TB* Thiene-Bassano thrust, *TC* Trento-Cles fault, *TL* Tonale line, *TR* Truden fault, *TRC* Travettore-Codevigo fault, *TRS* Tiers fault, *TT* Tremosine-Tignale thrust, *VBO* Val Bordaglia thrust, *VDS* Val di Sella thrust, *VS* Valsugana thrust, *VSA* Val Salata line, *VW* Villnöß/Val di Funes and Würzjoch/Passo del Erbe thrusts, *WA* Western Alps, *WS*A western Southern Alps. Map compiled from: faults attributed to phases (Castellarin & Cantelli, 2000), major units (Bigi et al., 1990; Schmid et al., 2004), Ladinian volcanics (Casetta et al., 2018), Dinaric flysch and Neoalpine flysch (Bigi et al., 1990; Caputo et al., 2010; Mellere et al., 2000), Jurassic basin-platform boundaries (Sieberer et al., 2023), Veneto Volcanic Province (Brombin et al., 2021)

**Fig. 2:**
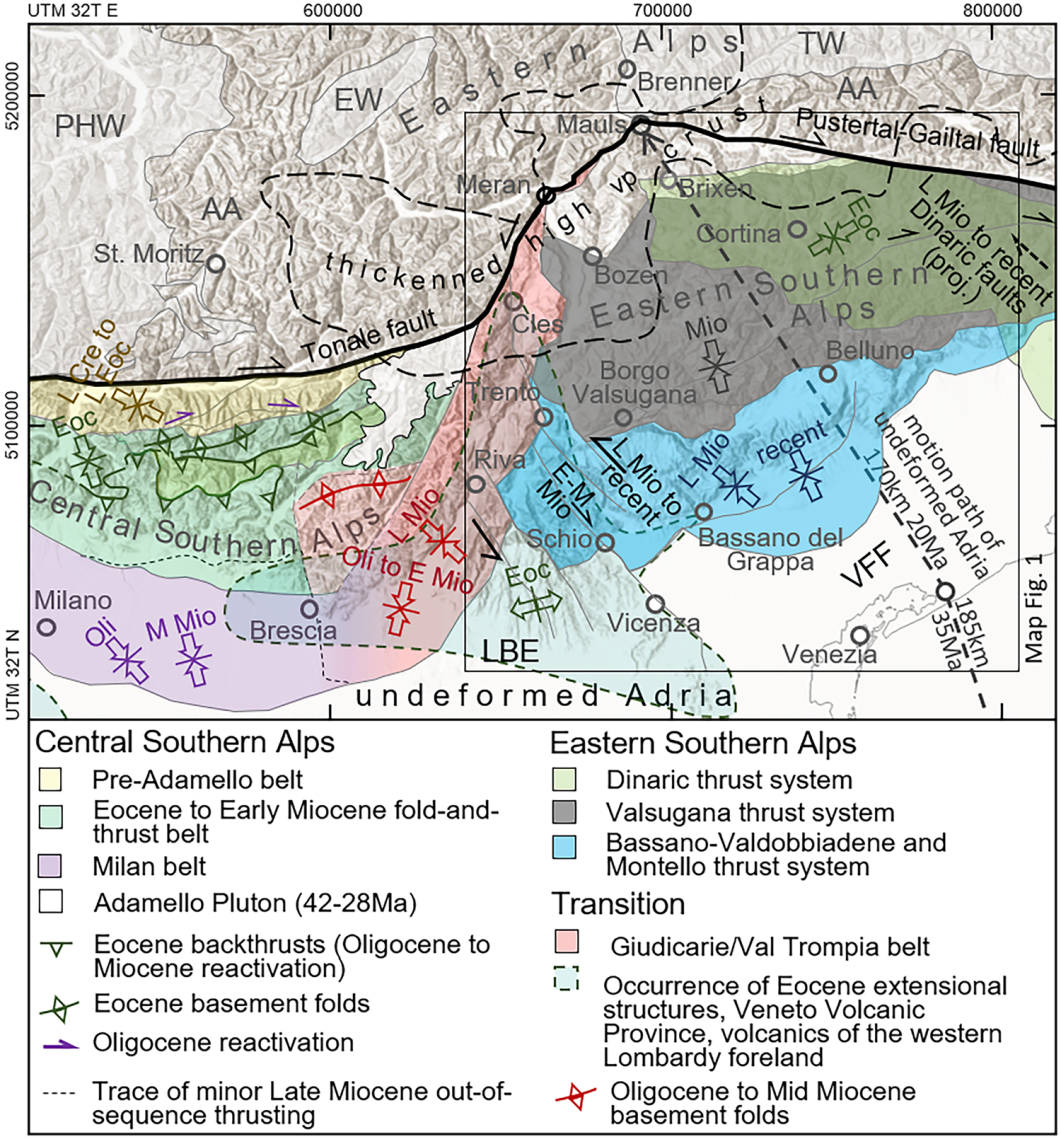
Deformation belts of the eastern and central Southern Alps and dominant stress field orientation. Delineated borders restrict domains of deformation and do not necessarily include piggyback offset areas. The southern boundary of the Pre-Adamello belt correspond to the cross sections of Schönborn (1992) and the thermochronological data of Zanchetta et al. (2015). Motion path of undeformed Adria with respect to fixed Europe is geometrically derived from motion paths of Ivrea and Apulia (Le Breton et al., 2021). Opposing arrows indicate dominant shortening direction (Caputo et al., 2010; Castellarin et al., 1992; Froitzheim et al., 1994; Picotti et al., 1995) at the specified chronostratigraphic period. Indicated zone of thickened crust with high p-wave velocity is following the interpretation of McPhee and Handy (2024) based on compiled low-earthquake-tomography and Moho models (Jozi Najafabadi et al., 2022; Spada et al., 2013). Epochs: *Cre* Cretaceous, *Eoc* Eocene, *Mio* Miocene, *Oli* Oligocene, *E* Early, *M* Middle, *L* Late. *AA* Austroalpine nappes, *BAS/MON* Bassano-Valdobbiadene and Montello thrust system, *DIN* Dinaric thrust system, *EW* Engadine Window, *GIU/VT* Giudicarie/Val Trompia belt, *LBE* Lessini-Bericci-Euganei block, *MIB* Milan belt, *PAB* Pre-Adamello belt, *PHW* Penninic and Helvetic nappes of the Western Alps, *TW* Tauern Window, *VFF* Veneto-Friuli foredeep, *VS* Valsugana thrust system

Polyphase convergence of the European plate and the Adriatic microplate (Adria) resulted in the closure of the interjacent Mesozoic oceanic basins and subsequent continental collision. Northern Adria was involved in both the Dinaric (predominantly Paleogene) and the Neoalpine (predominantly Neogene) orogenic phases. Both phases entail inversion of Adria and its Permo-Mesozoic passive margins (e.g., Handy et al., 2015; Schmid et al., 2008; van Hinsbergen et al., 2020). Dinaric shortening commenced at ~ 71–66 Ma, after the closure of the northern Neotethys and Sava oceanic basins east of Adria and was mainly characterized by southwest directed thrusting (Doglioni, 1987; Tari & Pamić, 1998; Ustaszewski et al., 2010). The Neoalpine collision of the northern Adriatic margin and Europe initiated at ~ 35–32 Ma and superseded the closure and south-directed subduction of the Piedmont-Liguria and Valais oceanic basins (e.g., Agard & Handy, 2021; Handy et al., 2010; Schmid et al., 1996) with Adria in upper-plate position (e.g., Handy & Oberhänsli, 2004; Laubscher, 1974, 1975; Schmid et al., 2004). Post-collisional northwest to north-directed indentation of Adria into the European lithosphere constitutes a key feature of the Neoalpine orogeny (e.g., Doglioni & Bosellini, 1987; Trümpy, 1973). Within the Neoalpine retro-wedge, sinistral offset along the Giudicarie fault system (GFS) delimited the emerging central Southern Alps (CSA) thick-skinned fold-and-thrust belt to the east. This allowed for an east–west decoupled retro-wedge evolution within Adria (Handy et al., 2015; Le Breton et al., 2021; Massari, 1990; van Hinsbergen et al., 2020), with the eastern Southern Alps (ESA) representing the eastern part (see Figs. 1 and 2).

The tectonic evolution of the ESA is traditionally constrained by a number of key observations: The sedimentary record indicates incipient offset along the Giudicarie fault system at 23–21 Ma (Luciani & Silvestrini, 1996; Sciunnach et al., 2010). Top ~ SSW directed in-sequence evolution of the ESA is recorded by syntectonic sediments only after the Langhian (Castellarin et al., 1987). A few low-temperature thermochronological data report regional Miocene cooling and a culmination of thrust related exhumation between 10 and 8 Ma (Dunkl et al., 1996; Eizenhöfer et al., 2023; Heberer et al., 2016; Reverman et al., 2012; Zattin et al., 2006). Upper Miocene syntectonic sediments provide evidence for in-sequence thrusting on the migrating ESA front, but indicate concurrent thrust activity rather than a distinct deformation transfer (Picotti et al., 2022). The absence of young detrital apatite fission-track data from the Southern Alpine molasse (Caputo et al., 2010; Monegato et al., 2010) suggests, that exhumation decreased to a minor rate in the Pliocene.

Apart from the Neoalpine record, Mesozoic low-temperature thermochronological data have been published by several authors (Dunkl et al., 2006; Dunkl et al., 1996; Emmerich et al., 2005; Pomella et al., 2012; Zattin et al., 2006; Zattin et al., 2003). A possible approach to handle this group was suggested by Dunkl et al. (2006). The schematic thermal history of the sampled Triassic volcanic rocks from the western Dolomites suggests a Late Triassic to Late Miocene residence between ~ 110 and 40 °C. Zattin et al. (2006) note that they frequently had to address the issue of mixed ages with subgroups and inherited single-grain ages within the apatite fission-track dataset. This suggests a prevalent low thermal overprint of the ESA, as full reset of thermochronological systems typically requires appropriate system conditions such as temperature and residence time (e.g., Carlson et al., 1999; Donelick et al., 1999; Ketcham, 2005; Vermeesch, 2019). In their studies, Tscherny et al. (2012) and Grobe et al. (2015) reconstruct the thermal history of the central ESA based on vitrinite reflectance data. Their findings indicate that temperatures at the base of the Permo-Mesozoic stratigraphic sequence were unevenly distributed, reaching a maximum of 135–150 °C. Published zircon (U-Th)/He data, collected along the TRANSALP transect, do not contain data related to the Neoalpine orogeny, with the exception of one sample from the vicinity of the Periadriatic fault (Eizenhöfer et al., 2021).

The approximate timing of the ESA tectonic evolution is hence constrained by well-defined boundary conditions, yet determined by only a few key observations and limited age constraints from geochronological data. These limitations preclude the development of a more sophisticated thermal and tectonic model. This study aims to constrain the Permian to Cenozoic thermal history and the Alpine exhumation pattern in the western ESA (Fig. 1). Since uplift is a major effect of collisional processes, a comprehensive thermochronological dataset can be used as an indicator of otherwise disguised or overprinted processes. To this end, low-temperature thermochronology has been used to extend the limited data set available and investigate the specific evolution of consistent tectonic blocks.

## Geological history and tectonic setting of the ESA

### The pre-Alpine geodynamic evolution

The metamorphic basement of the ESA is dominated by metasandstones and metapelites, deposited at least since the Cambrian with intercalations of metabasalt, metaporphyroid and partly graphitic shale (Kalvacheva et al., 1986). The Variscan metamorphic overprint ranges from lower greenschist facies in the Valsugana to amphibolite facies conditions in the Brixen area (Hammerschmidt & Stöckhert, 1987; Ring & Richter, 1994b). Post-Variscan Permian crustal thinning affected northern Adria, presumably related to the activity of a dextral transtensional, intra-Pangean mega shear zone (e.g., Cassinis et al., 2018; Cassinis et al., 1997; Doglioni, 1987; Muttoni et al., 2003; Pohl et al., 2018). Regional variations in subsidence and localized normal faulting consequently led to an initial morphological threshold-and-basin differentiation. Significant thickness variations in the Permian sediments of the affected area had a lasting impact on the ESA sedimentary and tectonic evolution (Cassinis et al., 1997; Doglioni, 1987; Massari & Neri, 1997). The extensional setting also allowed for the occurrence of the calc-alkaline volcanism of the Athesian Volcanic Group. Rottura et al. (1998) presume that the rocks are of a hybrid nature, resulting from the interaction between mantle-derived magmas and crustal materials. The authors attribute subduction-related trace element patterns to complex crustal compositions, rather than to the existence of a post-Variscan subduction zone during the early Permian. This is crucial when reconstructing the paleogeographic setting. Emplacement ages range from 290 to 274 Ma (Marocchi et al., 2008; Morelli et al., 2012; Visonà et al., 2007b). A very similar age range of 287–276 Ma is reported for the related intrusive bodies exhumed in the northern and southern zones of the ESA (Bargossi et al., 2010; Barth et al., 1993; Bellieni et al., 2010).

From the late Permian onward, transgression at the western margin of the Paleotethys gradually shifted the sedimentary facies from terrestrial to marine. This trend was interrupted only by Middle Triassic compression, which caused a first Mesozoic facies differentiation (e.g., Brandner & Gruber, 2019b; Carminati & Doglioni, 2022). The second, latest Anisian to Carnian facies differentiation was initiated by a break-up of the shallow-marine platform carbonates: Prograding carbonate platforms (Schlern Group), nucleating on morphological highs, were separated by open-sea intraplatform basins and evolved during subsidence on the Paleotethys shelf (e.g., Bosellini, 1984; Brandner & Gruber, 2019a, 2019b; van Hinsbergen et al., 2020). The timing of the Ladinian shoshonitic-basaltic and rhyolitic/andesitic lava flows as well as shallow plutonic bodies of Predazzo, Monzoni and Cima Pape, plus associated dikes and volcaniclastics, is well constrained. Intercalated tuff layers in Middle Triassic basin sediments of the Buchenstein Fm., Aquatona Fm., Zoppé Sandstone, and Wengen Fm. yield an age range of 242–238 Ma (Mundil et al., 1996; Storck et al., 2018).

Jurassic rifting and extension, attributed to the opening of the Piedmont-Liguria ocean, was the driver of the third major facies differentiation in the Southern Alps. Syn- and post-rift pelagic deposits constitute the sedimentary record of the basins (Lombardian, Belluno, and Slovenian basins), while condensed platform sediments represent the high zones (Trento and Friuli platforms, Julian high; Bernoulli & Jenkyns, 1974; Picotti & Cobianchi, 2017; Winterer & Bosellini, 1981). Picotti and Cobianchi (2017) report 80–100 km wavelength folding with an amplitude of ~ 800 m for the Friuli platform. They suggest that far-field compression occurred in the Late Jurassic as a result of conditional changes in the subduction of the northern Neotethys. Passive margin sedimentation and a decreasing rate of thermal subsidence characterized the Adriatic realm until the Early Cretaceous (e.g., Bertotti et al., 1993; Carminati et al., 2010; Fantoni & Scotti, 2003; Picotti et al., 1997; Winterer & Bosellini, 1981).

### The Alpine tectonic evolution

The tectonic response of the Adriatic crust to the Adria-Europe collision was predisposed by internal petrographic, sedimentological, and structural heterogeneities and the corresponding domains of disparate rheological properties (Masetti et al., 2012; Sieberer et al., 2023, 2024; Winterer & Bosellini, 1981). One pronounced domain boundary is represented by the transition between the Lombardian basin (west) and the Trento platform (east; e.g., Castellarin, 1972). In contrast to the basin stratigraphy, the platform sediments exhibit a thinner Permo-Mesozoic sedimentary succession, resting on the rocks of the Athesian Volcanic Group, which has a cumulative thickness of ~ 4 km (Marocchi et al., 2008). Their plutonic equivalents range in depth from shallow (e.g., Selli, 1998) to ~ 20 km (Castellarin et al., 2006a; Jozi Najafabadi et al., 2022) and have altered the crustal composition. The combination of a thin sedimentary cover and a block of rigid rock increases the resistance to deformation. Consequently, deformation is preferentially focused along inherited faults bounding reinforced regions (Sieberer et al., 2024). The western margin of the Trento platform roughly coincides with the position of the NNE-SSW trending GFS (Bertotti et al., 1993; Gregnanin et al., 2010; Verwater et al., 2021, and references therein). This crustal tear is oblique to the present northern boundary of the Adriatic crust, reaches down to the lower crust (Handy et al., 2015; Spada et al., 2013) and commenced in the latest Oligocene (Pomella et al., 2011; Pomella et al., 2012), at the latest from 23 to 21.5 Ma onwards (Castellarin et al., 2005; Luciani & Silvestrini, 1996; Scharf et al., 2013; Schmid et al., 2013; Sciunnach et al., 2010).

In this study, the term ‘Adriatic Indenter’ refers to Adriatic crust south of the Eoalpine orogen. This corresponds to the present-day leading edge of Adria, following the lithospheric model of Agard and Handy (2021), and is consistent with the lithosphere configuration beneath the Eastern Alps presented by McPhee and Handy (2024). The fold and thrust belts of the Southern Alps are considered accreted to the Neoalpine orogen, and are therefore separated from the indenting crust underneath the basal thrusts. From the latest Oligocene onwards, the leading edge of Adria to the west of the GFS is referred to as ‘Insubric Indenter’, while we employ the term ‘Dolomites Indenter’ for the indenting Adriatic leading edge to the east of the GFS (terminology of Frisch et al., 2000).

Northward movement of the Dolomites Indenter east of the GFS resulted in the updoming of the Tauern region (Bertrand et al., 2017; Fügenschuh et al., 1997; Rudmann et al., 2024, and references therein; Selverstone, 1988), piggyback top-north thrusting on the sub-Tauern ramp (Ortner et al., 2006; TRANSALP Working Group, 2002, correction published 2006), and eastward lateral escape (e.g., Favaro et al., 2017; Ratschbacher et al., 1991a; Ratschbacher et al., 1991b). However, Neoalpine shortening also affected the upper crust of the Dolomites Indenter. 25–35 km shortening accumulated between Cles and Bassano del Grappa (Selli, 1998; Verwater et al., 2021; see Fig. 1 for geographic reference) and at least 35–46 km along the TRANSALP transect between Toblach and Treviso (Castellarin et al., 2006a; Kummerow, 2003; McPhee & Handy, 2024). This shortening has been accommodated in the Miocene to still ongoing formation of the ESA fold-and-thrust belt (Castellarin et al., 1992). The evolution of the ESA is mainly constrained by the sedimentary record: A geometric interpretation of wedge shaped stratigraphic units, located at the ESA frontal thrust system, recorded a synorogenic foreland flexure enhancement from the Chattian to the Pleistocene (Picotti et al., 2022). The “late-syntectonic Valsugana molasse” of Castellarin et al. (1992) proofs Serravallian to Tortonian exhumation, erosion, and synkinematic deposition. Southward prograding, clastic delta and fan sediments and associated growth structures testify to folding and thrusting, exposure, and erosion of the ESA frontal high zone since at least 8–6 Ma (Picotti et al., 2022, and references therein). Detrital fission-track and petrographic provenance analyses suggest a drainage divide shift from north of the Periadriatic fault to within the evolving, south verging, ESA fold-and-thrust belt in the Serravallian (Stefani et al., 2007). At the latest during the Messinian, the catchment area of the ESA drainage system emerged to its present state (Monegato et al., 2010). These constraints emphasize a Middle to Late Miocene major tectonic pulse. Existing low-temperature thermochronology data likewise indicate a phase of rapid ESA fold-and-thrust belt evolution in the Late Miocene (Dunkl et al., 1996; Heberer et al., 2016; Zattin et al., 2006). Nevertheless, some of the few published time–temperature models indicate, that rapid exhumation may have commenced as early as the late Oligocene (Zattin et al., 2006). Also, Curzi et al. (2024) infer persisting activity of WSW-ENE trending thrusts from at least the Oligocene to the Late Miocene based on U–Pb formation ages of tectonic carbonates.

The polyphase nature of Alpine compressional tectonics is recorded by syntectonic sediments, the orientation of tectonic structures, and fault slip data. Paleostress analyses allow the assignment of tectonic structures to orogenic phases (e.g., Caputo et al., 2010; Castellarin et al., 1998; Castellarin & Vai, 1986; Castellarin et al., 2006b; Doglioni, 1987; Massari, 1990), which correspond to belts of focused shortening and exhumation (Fig. 2). The following section provides a concise overview of applicable tectonic phases.

1. Pre-Adamello (Late Cretaceous, sealed at 42 Ma): This phase comprises the formation of a structural system partly related to Eoalpine nappe stacking (e.g., Zanchi et al., 1990). The Pre-Adamello belt is an E-W to NE-SW striking thick-skinned fold-and-thrust belt and records ~ NW–SE compression. Its structural evolution is post-dated by the first magmatic pulse of the Eocene to early Oligocene Adamello intrusion at ~ 42 Ma (Brack, 1981; De Sitter & De Sitter-Koomans, 1949; Del Moro et al., 1983; Hansmann & Oberli, 1991; Zanchetta et al., 2015). There is no obvious prolongation of Pre-Adamello structures east of the Giudicarie belt visible in the mapping and structural analysis data (Castellarin & Vai, 1986; Doglioni, 1987).
2. Dinaric (Eocene): The tectonic system of NW–SE striking folds and thrusts with ~ top-southwest kinematics is related to the Dinaric collision and evolved concurrently to thick-skinned thrusting in the Dinaric orogen (Cousin, 1981; Doglioni, 1985; Doglioni, 1987). However, the Dinaric and the Alpine front were likely to be kinematically decoupled by a system of transfer faults. Handy et al. (2015) therefore consider the Dinaric phase thrusts of the ESA as Alpine backthrusts. Shortening is estimated to be at least 10 km (Doglioni, 1987). Doglioni and Bosellini (1987) limit the occurrence of Dinaric structures in the ESA to a NW–SE striking front, directly west of Brixen in the north and Belluno in the south.

It is worth noting that the tectonic structures labelled as “Dinaric trends” that are observed near Brescia, Riva del Garda, the Non Valley, and Trento are assigned to the Chattian – Burdigalian based on the sedimentary record (Castellarin et al., 1992; Castellarin et al., 1987; Castellarin et al., 2005). These structures are therefore primarily considered to be Eocene faults that were reactivated during Neoalpine shortening within a local stress field (Castellarin et al., 2005). Eocene extension at the southern Trento platform is well documented and was accompanied by the emplacement of the Veneto Volcanic Province (e.g., Barbieri et al., 1991; Zampieri, 1995). Fantoni et al. (1999) identified intercalated volcanic layers within the Eocene sedimentary sequence to the south of the Alpine foothills in the Brescia region, attributing them to the same volcanic district. To the southwest of Milano, the same authors documented Eocene NW–SE trending normal faults associated with volcanics, buried under Po Plain foreland sediments. The extensional described formed to the southwest of the emerging Dinaric structures of the ESA (Doglioni & Bosellini, 1987) and to the south of the evolving Eocene fold-and-thrust belt of the CSA (Zanchetta et al., 2015) (Fig. 2).

(3) Insubric-Helvetic/Gonfolite (Chattian–Langhian): The ~ 35 Ma Neoalpine collision did not affect the ESA immediately as deformation was dominantly accumulated by shortening, exhumation, and lateral extrusion north of the Periadriatic fault system (e.g., Castellarin et al., 2006a; Ortner et al., 2006; Ratschbacher et al., 1991a). Provenance analyses based on detrital fission-track data and petrographic analyses of Monegato et al. (2010) suggest Chattian to Burdigalian uplift in the northernmost ESA. Structural evidence for a post-collisional NNE-SSW directed reorientation of the stress field from the late Oligocene to the Early Miocene was compiled by Caputo et al. (2010). Corresponding shortening has also been reported for the western part of the Villnöß/Val di Funes and Würzjoch/Passo del Erbe thrusts (Ring & Richter, 1994a).
(4) Giudicarie (~ 23–9? Ma): Scharf et al. (2013) constrained incipient sinistral transpressive Giudicarie kinematics to ~ 23–21 Ma. The onset was recorded by the youngest marine sediments within the Monte Brione Fm. east of Riva del Garda post-dated by deformation (Luciani & Silvestrini, 1996), and the synorogenic Monte Orfano conglomerate (Sciunnach et al., 2010) which is assigned to the kinematically linked Val-Trompia belt (Laubscher, 1988; Picotti et al., 1995). ^40^Ar/^39^Ar pseudotachylyte dating documents Early Miocene slip (17 Ma) on the Passeier fault and Oligocene (32 Ma) as well as Early Miocene (17 Ma) slip on the Northern Giudicarie fault (Müller et al., 2001; Pleuger et al., 2012). Early Miocene incipient rapid updoming and exhumation of the Tauern Window is coupled to Giudicarie activity and accommodated the majority of Neoalpine shortening in combination with eastward lateral escape (Scharf et al., 2013; Schmid et al., 2013). High exhumation rates due to Tauern Window unroofing and footwall exhumation along the Brenner shear zone were recorded between 20 and 15 Ma (Fügenschuh et al., 1997), faulting at the Brenner normal fault ceased at 9 Ma (Wolff et al., 2021).
(5) Valsugana (~ 14–8 Ma): Most of the ESA exhumation is associated with SSE-directed shortening at the Valsugana thrust system (e.g., Doglioni, 1987; Doglioni, 1992b; Picotti et al., 2022). Heberer et al. (2016) infer a shift from Oligocene and Early Miocene Tauern Window exhumation in front of the Dolomites Indenter to Middle and Late Miocene coupled state Tauern Window and ESA exhumation. Their model is based on the Tauern Window exhumation model of Bertrand et al. (2015) and thrust-focused thermochronological data from the ESA. The authors follow the assumption of a fast deformation transfer into the ESA due to a critical tight fold geometry of the Tauern Window. The exhumation and cooling related rheologic lock of the Tauern Window shortening is also supported by the kinematic restoration model of Rudmann et al. (2024). CSA – ESA decoupling ceased due to the joint activity of the Valsugana thrust system and the Milan belt (Fig. 2), which persisted until the early Pliocene (Picotti et al., 1995). Castellarin et al. (1987) suggested a post-Langhian (~ 14 Ma) earliest onset of Valsugana thrusting, based on the youngest undeformed footwall sediments of Valsugana. Reverman et al. (2012) obtained a 10–8 Ma fast exhumation phase as a last pulse of the Valsugana phase from the inversion of thermochronological data.
(6) Adriatic/Bassano (~ 10–4 Ma): A significant deformation shift occurred in the Late Miocene. The southward progression of the ESA front enabled the evolution of a deeper-seated, thick-skinned thrust system parallel and interconnected to the Valsugana thrust system (Castellarin et al., 2006a; Curzi et al., 2023), allowing for reactivation pulses of the latter. The emerging Bassano-Valdobbiadene thrust system (Fig. 2; Picotti et al., 2022; Montello thrust system of Castellarin and Cantelli, 2000) has no prolongation towards the west where it is bordered by a set of NW–SE striking, tear fault like strike-slip faults. The sinistrally reactivated Schio-Vicenza fault system and related faults (Fig. 2; for a discussion of controversial sense of shear see Zampieri et al., 2021) still decouple the ESA to the west (Castellarin et al., 1998). Dextral reactivation of NW–SE trending Dinaric faults, the Fella-Sava fault, and parts of the Valsugana thrust system ensures the decoupling of the ESA to the east (Castellarin et al., 1992; Grützner et al., 2021; Heberer et al., 2016; Moulin et al., 2016; Serpelloni et al., 2016).
(7) Frontal thrust system (ongoing): The southernmost thrusts of the ESA evolved during the Pliocene and the Pleistocene. The ~ NNW-SSE shortening direction deviates from the general progressive counterclockwise trend (Caputo et al., 2010). Most authors refer to the frontal thrust system as an in-sequence branch of the Bassano-Valdobbiadene thrust system (e.g., Castellarin et al., 1998; Doglioni, 1992a; Picotti et al., 2022; Schönborn, 1999).

### Synorogenic magmatic events

Magmatic events have the capacity to reset or perturb the regional thermochronological record. Consequently, their spatial and temporal occurrence is of crucial importance for the interpretation of temperature-related data. Three periods of Maastrichtian to Cenozoic magmatism can be distinguished within the ESA:

*Maastrichtian to late Eocene alkaline ultrabasic magmatism* in the ESA is corroborated by the existence of lamprophyric and lamproitic dikes and most likely related to extension due to the east-directed Adria subduction in the Dinarides. The dikes yield Maastrichtian (~ 71–67 Ma), to late Eocene age (~ 34 Ma; Galassi et al., 1994; Lucchini et al., 1983). Their occurrence is very regional and does not encourage a large-scale thermal imprint (Narduzzi et al., 2023).

*Eocene to Oligocene Periadriatic magmatism:* Pre- to syn-collisional Paleogene magmatic rocks exhibit emplacement ages between 42 and 26 Ma (Kästle et al., 2020; Pomella et al., 2011 and references therein) and trace the Periadriatic fault system from Biella at the Canavese line segment in the west to the Karawanken mountains along the Pustertal-Gailtal fault segment in the east. Handy et al. (2015) propose an evolving tear in the European slab at ~ 35 Ma, propagating to the west and resulting in asthenospheric upwelling, melting and magma migration. However, this timing does not align with the age of the first plutons, as noted by Ji et al. (2019). Instead, they propose that enhanced corner flow, resulting from the steepening of the still intact European slab, triggered the Periadriatic magmatism. Within the Southalpine realm the Adamello batholith is the only major magmatic body related to this event (Fig. 1). The associated thermal perturbation is confined to the vicinity of the plutonic bodies. However, Visonà et al. (2022) present evidence of a “hidden intrusion” in the Brixen area with an emplacement age of 35–28 Ma.

*Middle Eocene to Early Miocene Veneto Volcanic Province magmatism:* The intraplate magmatism of Val d’Adige (42–41 Ma), Lessini Mountains (45–39 Ma), Marostica Hills (32–22 Ma), and Euganean Hills (32–28 Ma) obviously coincides with the Periadriatic magmatism (Brombin et al., 2021). A common link to the European subduction is therefore assumed (e.g., Handy et al., 2015). However, the geochemical signature of the Veneto Volcanic Province suggests metasomatic enrichment within the Adriatic crust during decompressional melting and magmatic ascent (Brombin et al., 2021). Based on this model, thermal overprinting of the Adriatic lithosphere is limited to the vicinity of the magmatic pathways.

## The crustal structure of the ESA fold-and-thrust belt

**Fig. 3:**
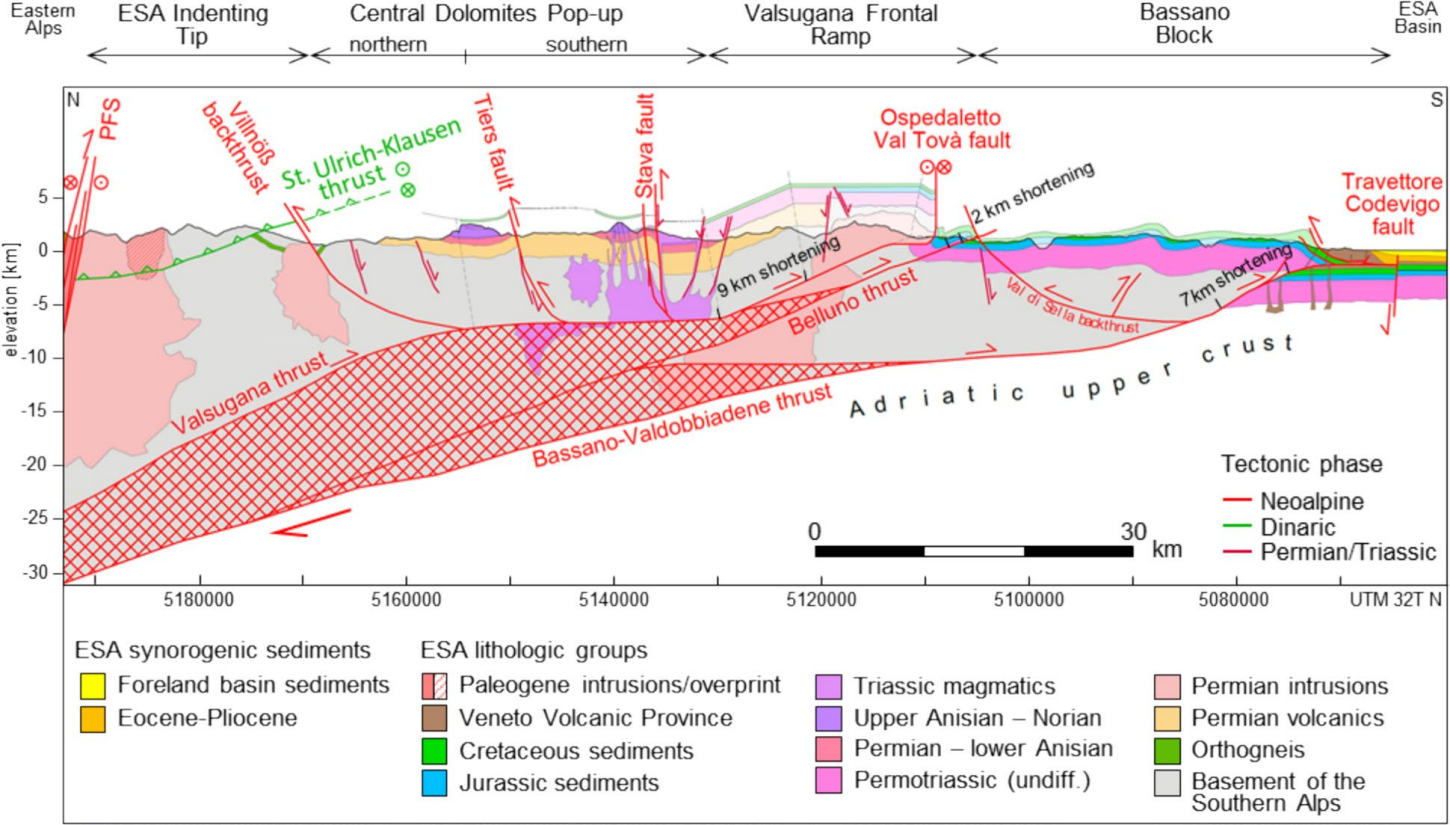
Geological cross section between Mauls and Bassano del Grappa (section trace indicated in Fig. 1). Four fault-bound tectonic blocks of consistent Neoalpine behavior are indicated at the top. The geometry of crustal scale thrusts is deduced from hinterland strata dip values at the surface. An interlinked system of transfer faults must be assumed between the major thrust systems at depth. The cross section is based on maps and in part adapted from compiled sections (Baggio et al., 1969; Barbieri & Grandesso, 2007; Brandner et al., 2019; Dal Piaz et al., 1970; Nardin et al., 1972; Reiter et al., 2018; Rossi et al., 1977; Sassi et al., 1970; Selli, 1998)

The ESA show their greatest orogen-perpendicular extent between Mauls at the northernmost kink of the Periadriatic fault system and Treviso, where the southeastward step of the frontal thrust system is largest (Fig. 1). To the west, the Meran-Mauls fault, the Giudicarie fault system, and the Trento-Cles/Schio-Vicenza strike-slip zones limit further extension of the ESA structures (e.g., Doglioni, 1987; Pola et al., 2014; Pomella et al., 2011). Towards the east, the ESA become progressively narrower, delimited by the Periadriatic fault and the Dinaric orogen, and are tapered off by the Mid-Hungarian fault zone (Schmid et al., 2008). Figure 3 illustrates a ~ N-S cross section from Mauls to Bassano del Grappa. The geometry of frontal thrusts, backthrusts, deep-seated ramps and flat sections is mainly constrained by compiled dip values and lithologic boundaries (Baggio et al., 1969; Barbieri & Grandesso, 2007; Brandner et al., 2019; Dal Piaz et al., 1970; Nardin et al., 1972; Rossi et al., 1977; Sassi et al., 1970; Selli, 1998), and the expected kinematics, considering the timing of thrusting as discussed in Sect. 2.2. For the interpretation of the new thermochronological data (Sect. 6), we will refer to consistent tectonic blocks, separated by distinct thrusts or tectonic displacements, as indicated at the top of Fig. 3. Balancing of the cross section indicates a total minimum shortening of only ~ 18 km, which is less than the estimates provided by Selli (1998; northern Giudicarie fault to Bassano), Castellarin et al., (2006a; TRANSALP transect), and Moulin and Benedetti (2018; Friuli area).

## Methods

Low-temperature thermochronology samples were collected from exposed bedrock that showed minimal degrees of weathering. Sample preparation follows that of Kohn et al. (2019). Apatite fission-track (AFT) analyses were undertaken using the external detector method at the Department of Geology, University of Innsbruck. Sample irradiation was carried out at the Oregon State University Radiation Center research reactor. Single-grain (U-Th)/He analyses on apatites (AHe) and zircons (ZHe) were performed at the Low-Temperature geochronology laboratory of the Geoscience Center at the University of Göttingen. To retrace the thermal history of the samples, an inversion of the AFT and AHe data was carried out using the thermal inversion routine implemented in HeFTy v2.1.7 (Ketcham, 2005, 2024; Ketcham et al., 2007). Modelled cooling paths were statistically further refined by producing a graphical mean contour map. Further details on sample preparation, data acquisition and modelling are given in the Appendix, Sect. 1.

## Thermochronological data

We present 22 apatite fission-track (AFT) data, ranging from 181 to 19 Ma (Table 1). Six AFT data do not pass the χ^2^ test and, therefore, require careful handling with respect to overfitting. In particular, it is essential to conduct a detailed examination of the data to ascertain whether there are any indications of continuous age ranges or multimodal age distributions (Galbraith, 2005; Vermeesch, 2019). Single-grain age distribution plots are, therefore, used in addition to the radial plots in this study. For eight samples, a sufficient number of track lengths were measured to allow for inverse time–temperature modelling. All single-grain age plots and radial plots are provided in the Appendix, Sect. 2.

**Table 1:** Apatite fission-track data from the western ESA

Sample	Lithology	Location				Apatite analysis									
		UTM 32 T E [m]	UTM 32 T N [m]	Z [m]	locality	Count crystals	Ns	rs	Ni	ri	rd	U [ppm]	P(χ) [%]	Age [Ma]	± 1 s [Ma]
TKP002	Athesian Volcanic Group	692563	5128150	995	Cermis	20	993	0.157	447	0.071	5.694	0.19	81.21	**156.5**	**13.0**
TKP003	Triassic intrusives	701255	5131982	1054	Predazzo	28	172	0.014	602	0.049	5.772	0.12	64.61	**20.9**	**2.3**
TKP004	Triassic intrusives	703408	5132204	1190	Passo Rolle, Zaluna	51	1791	0.070	734	0.029	5.850	0.06	80.99	**176.3**	**13.2**
TKP005	Athesian Volcanic Group	675707	5133837	230	Auer, toll station	25	2065	0.300	1215	0.177	5.928	0.44	0.00	**138.7**	**11.3**
TKP006	Athesian Volcanic Group	678133	5137084	244	Auer, Eurocenter	18	256	0.043	369	0.061	6.006	0.16	8.46	**53.8**	**6.7**
TKP007	Athesian Volcanic Group	687480	5151876	360	Blumau	29	945	0.053	710	0.040	6.084	0.10	0.01	**97.9**	**7.0**
TKP008	Athesian Volcanic Group	692174	5153525	730	Prösels	46	1673	0.092	1017	0.056	6.240	0.12	0.00	**131.0**	**8.7**
TKP009	Buchenstein-Fm	701055	5160131	2005	Pufels, Fillner Kreuz	31	592	0.021	560	0.020	6.319	0.04	15.91	**80.3**	**7.5**
TKP012	Werfen-Fm	701961	5160646	1563	Pufels, geotrail	20	449	0.183	564	0.229	6.397	0.48	0.17	**73.0**	**7.8**
TKP013	Gröden-Fm	702588	5161113	1178	St. Ulrich, Runggaditsch	17	359	0.048	790	0.106	6.475	0.23	0.57	**41.9**	**7.7**
TKP017	Southalpine crystalline basement	708785	5174835	2419	Plosehütte	15	137	0.041	241	0.072	6.865	0.13	100.00	**48.7**	**6.0**
TKP018	Southalpine crystalline basement	706771	5172484	1625	Palmschoss	37	777	0.040	1684	0.087	7.021	0.17	89.90	**40.4**	**3.0**
TKP021	Southalpine crystalline basement	704560	5175334	998	St. Andrä	29	226	0.023	505	0.050	7.255	0.09	94.46	**40.5**	**4.1**
TKP028	Permian intrusives	701088	5116970	2848	Cima d'Asta, summit	24	791	0.066	2318	0.193	7.411	0.37	15.81	**31.8**	**2.5**
TKP029	Permian intrusives	700815	5116096	2344	Cima d'Asta, South	48	842	0.040	3908	0.185	7.489	0.33	37.90	**20.2**	**1.5**
TKP030	Permian intrusives	700299	5115369	1864	Cima d'Asta, South	33	346	0.029	1805	0.150	7.567	0.25	7.89	**19.0**	**1.8**
TKP034	Southalpine crystalline basement	698443	5106313	906	Bieno	45	150	0.007	83	0.004	7.645	0.01	100.00	**170.7**	**25.6**
TKP037	Southalpine crystalline basement	688890	5098040	894	Val di Sella, Colle Stanga	30	1431	0.146	789	0.081	7.802	0.16	49.27	**181.2**	**15.1**
TKP040	Mafic dykes (Veneto Volcanic Province?)	671391	5078425	1223	Passo Borcola	19	793	0.133	842	0.141	7.880	0.25	0.45	**103.1**	**6.5**
TKP042	Buchenstein-Fm	696911	5135049	2086	Passo Feudo, geotrail	50	2011	0.060	1176	0.035	7.958	0.06	88.79	**168.2**	**12.0**
TKP052	Val Lagarina Basalts	699768	5069894	410	Schio, Salcedo	25	593	0.057	1724	0.164	8.192	0.26	89.21	**35.3**	**2.8**
TKP053	Val Lagarina Basalts	681125	5059067	604	Passo Zovo, Valdagno	70	474	0.012	1157	0.029	8.270	0.05	100.00	**42.3**	**3.5**

Track density r [× 10^5^ tracks/cm^2^]. Track count N [tracks]. Apatite fission-track ages (bold emphasis) were calculated using a Zeta of 250,42 ± 14,79 and dosimeter glass IRMM540

Eight apatite (U-Th)/He (AHe) data, spanning a range of 20–7 Ma, were processed (Table 2). Sample specific single-grain eU and spherical radius versus date plots are provided in the Appendix, Sect. 2. The majority of the samples share common parameter correlations as summarized by Flowers et al. (2022).

**Table 2:** Apatite (U-Th-Sm)/He data from the western ESA

Sample	Lithology	Location				Grain	He		U238			Th232			Sm			Th/U	Ejection	Uncorr	Ft-corr		Unweighted	
		UTM 32 T E [m]	UTM 32 T N [m]	Z [m]	Locality	ID	Vol. [ncc]	± 1σ [%]	Mass [ng]	± 1σ [%]	Conc. [ppm]	Mass [ng]	± 1σ [%]	Conc. [ppm]	Mass [ng]	± 1σ [%]	Conc. [ppm]	Ratio	Corr. (Ft)	He age [Ma]	He age [Ma]	± 2σ [Ma]	Aver. [Ma]	± 1σ [Ma]
TKP002	Athesian Volcanic Group	692563	5128150	995	Cermis	a1	0.187	1.6	0.060	2.0	13	0.136	2.4	30	2.07	4.5	461	2.3	0.727	14.4	19.8	1.8	**19.5**	**2.9**
						a2	0.113	1.8	0.047	2.1	21	0.119	2.4	54	1.64	4.5	740	2.5	0.749	10.7	14.3	1.3		
						a3	0.302	1.5	0.074	1.9	30	0.196	2.4	79	2.23	4.5	897	2.6	0.749	18.3	24.4	2.1		
TKP009	Buchenstein Fm	701055	5160131	2005	Pufels, Fillner Kreuz	a1	0.235	1.5	0.056	2.0	7	0.315	2.4	42	1.96	1.7	260	5.7	0.809	13.6	16.8	1.2	**13.8**	**1.1**
						a2	0.096	1.7	0.025	2.8	6	0.157	2.4	40	2.01	1.7	509	6.3	0.749	10.4	13.8	1.2		
						a3	0.095	1.7	0.028	2.7	7	0.226	2.4	54	2.40	1.7	569	8.2	0.698	8.0	11.4	1.2		
						a4	0.076	1.7	0.017	3.6	11	0.227	2.4	153	0.96	1.7	648	13.7	0.629	8.3	13.3	1.6		
TKP028	Permian intrusives	701088	5116970	2848	Cima d'Asta, summit	a1	0.100	1.6	0.073	1.9	36	0.123	2.4	60	1.49	1.7	723	1.7	0.745	7.3	9.8	0.9	**12.0**	**1.2**
						a2	0.265	1.5	0.134	1.8	29	0.183	2.4	40	2.90	1.7	636	1.4	0.812	11.0	13.6	0.9		
						a3	0.142	1.7	0.116	1.9	70	0.086	2.4	52	1.31	1.7	788	0.7	0.635	8.0	12.7	1.5		
TKP029	Permian intrusives	700815	5116096	2344	Cima d'Asta, South	a1	0.493	1.5	0.358	1.8	31	0.549	2.4	48	6.71	1.7	585	1.5	0.822	7.6	9.3	0.6	**12.9**	**2.5**
						a2	0.053	2.0	0.070	2.0	60	0.056	2.4	48	0.83	1.7	712	0.8	0.556	4.9	8.9	1.3		
						a3	0.419	1.5	0.257	1.8	48	0.143	2.4	27	2.75	1.7	510	0.6	0.783	11.1	14.2	1.1		
						a4	0.764	1.4	0.261	1.8	39	0.519	2.4	77	5.04	1.7	746	2.0	0.780	15.1	19.3	1.5		
TKP030	Permian intrusives	700299	5115369	1864	Cima d'Asta, South	a1	0.213	1.6	0.182	1.8	45	0.273	2.4	67	3.30	4.5	812	1.5	0.760	6.5	8.6	0.7	**9.5**	**0.6**
						a2	0.160	1.6	0.154	1.8	59	0.166	2.4	63	2.04	4.5	779	1.1	0.689	6.4	9.2	0.9		
						a3	0.420	1.5	0.331	1.8	79	0.239	2.4	57	3.20	4.5	768	0.7	0.762	8.5	11.1	0.9		
						a4	0.144	1.6	0.126	1.9	39	0.159	2.4	49	2.15	4.5	663	1.3	0.742	6.6	9.0	0.8		
TKP042	Buchenstein Fm	696911	5135049	2086	Passo Feudo, geotrail	a1	0.095	1.7	0.033	2.4	8	0.095	2.4	24	1.59	1.7	394	2.9	0.760	11.8	15.5	1.3	**15.0**	**1.7**
						a2	0.048	1.9	0.012	5.7	6	0.076	2.4	37	0.45	1.7	221	6.4	0.621	12.1	19.5	2.5		
						a3	0.048	1.9	0.015	3.9	6	0.087	2.4	32	0.51	1.7	188	5.7	0.748	10.3	13.8	1.3		
						a4	0.061	1.9	0.024	2.8	8	0.117	2.4	40	1.06	1.7	362	4.8	0.745	8.4	11.3	1.0		
Do-534	Permian intrusives	710570	5115409	760	Canale San Bovo	a1	0.055	2.6	0.081	2.0	37	0.092	2.4	42	1.75	3.7	790	1.1	0.616	3.9	6.4	0.8	**7.3**	**0.7**
						a2	0.031	3.2	0.049	2.2	53	0.043	2.5	47	0.65	3.7	701	0.9	0.605	3.9	6.4	0.9		
						a3	0.016	4.7	0.013	4.9	11	0.020	2.7	17	0.71	3.7	600	1.5	0.616	5.8	9.3	1.5		
						a4	0.046	2.9	0.059	2.1	34	0.061	2.5	36	1.16	3.7	675	1.0	0.670	4.6	6.9	0.8		
Do-537	Permian intrusives	684221	5100971	594	Marter	a1	0.009	5.3	0.015	5.1	14	0.028	2.8	26	0.42	3.7	394	1.9	0.480	3.1	6.5	1.3	**8.6**	**1.6**
						a2	0.122	1.8	0.072	2.0	18	0.166	2.4	41	2.08	3.7	517	2.3	0.741	7.8	10.6	1.0		
						a3	0.011	5.5	0.020	3.5	18	0.033	2.5	30	0.50	3.7	455	1.6	0.538	3.0	5.5	1.0		
						a4	0.038	3.0	0.025	3.1	12	0.047	2.5	22	0.84	3.7	393	1.9	0.596	7.2	12.0	1.7		

AHe data are reported as unweighted average (bold emphasis). He volume for standard temperature and pressure; Correction factor for alpha‐ejection according to Farley et al., 1996 and Hourigan et al., 2005; Single-grain age uncertainty includes analytical and estimated uncertainty. Analysis parameters, modelling and concentration calculations, and quality parameters of the measurement see Appendix, Sect. 2. *aver.* average, *conc.* concentration, *corr.* corrected/correction, *uncorr.* uncorrected, *vol.* volume

Five zircon (U-Th)/He (ZHe) samples were processed (Table 3). These samples originate from the Southalpine crystalline basement and the Permian magmatics.

**Table 3:** Zircon (U-Th-Sm)/He data from the western ESA

Sample	Lithology	Location				Grain	He		U238			Th232			Sm			Th/U	Ejection	Uncorr	Ft-corr		Unweighted	
		UTM 32 T E [m]	UTM 32 T N [m]	z [m]	Locality	ID	Vol. [ncc]	± 1σ [%]	Mass [ng]	± 1σ [%]	Conc. [ppm]	Mass [ng]	± 1σ [%]	Conc. [ppm]	Mass [ng]	± 1σ [%]	conc. [ppm]	Ratio	Corr. (Ft)	He age [Ma]	He age [Ma]	± 2σ [Ma]	Aver. [Ma]	± 1σ [Ma]
TKP018	Southalpine crystalline basement	706771	5172484	1625	Palm-schoss	a1	11.663	1.5	0.642	1.8	154	0.402	2.4	97	0.03	2.4	7	0.6	0.767	130.4	169.9	14.0	**222.9**	**9.0**
						a2	12.969	1.5	0.557	1.8	106	0.251	2.4	48	0.02	2.4	4	0.5	0.780	172.6	221.5	17.6		
						a3	20.770	1.5	0.843	1.8	228	0.289	2.4	78	0.03	2.4	9	0.3	0.757	186.5	246.5	21.1		
						a4	18.915	1.5	0.619	1.8	220	0.833	2.4	296	0.05	2.4	19	1.3	0.754	191.5	253.8	21.5		
TKP037	Southalpine crystalline basement	688890	5098040	894	Val di Sella, Colle Stanga	a1	64.118	1.5	2.118	1.8	351	0.648	2.4	107	0.07	2.4	11	0.3	0.764	229.9	301.0	25.1	**304.9**	**2.5**
						a2	73.224	1.5	2.313	1.8	394	0.479	2.4	82	0.03	2.4	6	0.2	0.792	245.2	309.6	23.8		
						a3	75.847	1.5	2.206	1.8	208	0.974	2.4	92	0.08	2.4	7	0.4	0.833	253.4	304.2	20.3		
6–47	Permian intrusives	700777	5117059	2830	Cima d'Asta	a1	106.325	1.6	6.021	1.8	778	2.361	2.4	305	0.06	24.3	7	0.4	0.775	132.4	170.9	14.0	**158.8**	**15.1**
						a2	109.283	1.6	7.963	1.8	679	3.087	2.4	263	0.11	24.4	9	0.4	0.802	103.2	128.7	9.7		
						a3	118.812	1.6	6.105	1.8	590	2.680	2.4	259	0.22	24.4	21	0.4	0.816	144.3	176.8	12.7		
9–17	Athesian Volcanic Group	679722	5153608	425	Auer	a1	62.901	1.6	2.217	1.8	268	1.364	2.4	165	0.06	25.0	7	0.6	0.773	201.7	261.1	21.4	**264.9**	**14.7**
						a2	91.668	1.6	3.399	1.8	376	2.078	2.4	230	0.11	24.9	12	0.6	0.795	192.0	241.5	18.4		
						a3	82.308	1.6	2.708	1.8	434	1.198	2.4	192	0.05	25.4	8	0.4	0.766	223.6	292.1	24.5		
Do-502	Southalpine crystalline basement	716363	5124498	1246	Passo Rolle South	a1	21.937	1.0	1.143	1.8	616	0.271	2.4	146	0.02	4.8	9	0.2	0.711	148.6	209.0	19.8	**181.6**	**17.6**
						a2	22.572	0.9	2.015	1.8	693	0.559	2.4	192	0.02	4.8	6	0.3	0.754	86.4	114.6	9.5		
						a3	13.787	1.0	0.651	1.8	129	0.314	2.4	62	0.02	4.8	3	0.5	0.767	155.4	202.7	16.1		
						a4	18.056	1.0	1.140	1.8	714	0.297	2.4	186	0.02	4.8	13	0.3	0.687	122.3	177.9	18.0		
						a5	35.736	1.0	1.765	1.8	525	0.875	2.4	260	0.03	4.8	9	0.5	0.728	148.2	203.5	18.2		

ZHe data are reported as unweighted average (bold emphasis). He volume for standard temperature and pressure; Correction factor for alpha‐ejection according to Farley et al., 1996 and Hourigan et al., 2005; Single-grain age uncertainty includes analytical and estimated uncertainty. Analysis parameters, modelling and concentration calculations, and quality parameters of the measurement see Appendix, section 2. *aver.* average, *conc.* concentration, *corr.* corrected/correction, *uncorr.* uncorrected, vol. volume

**Fig. 4:**
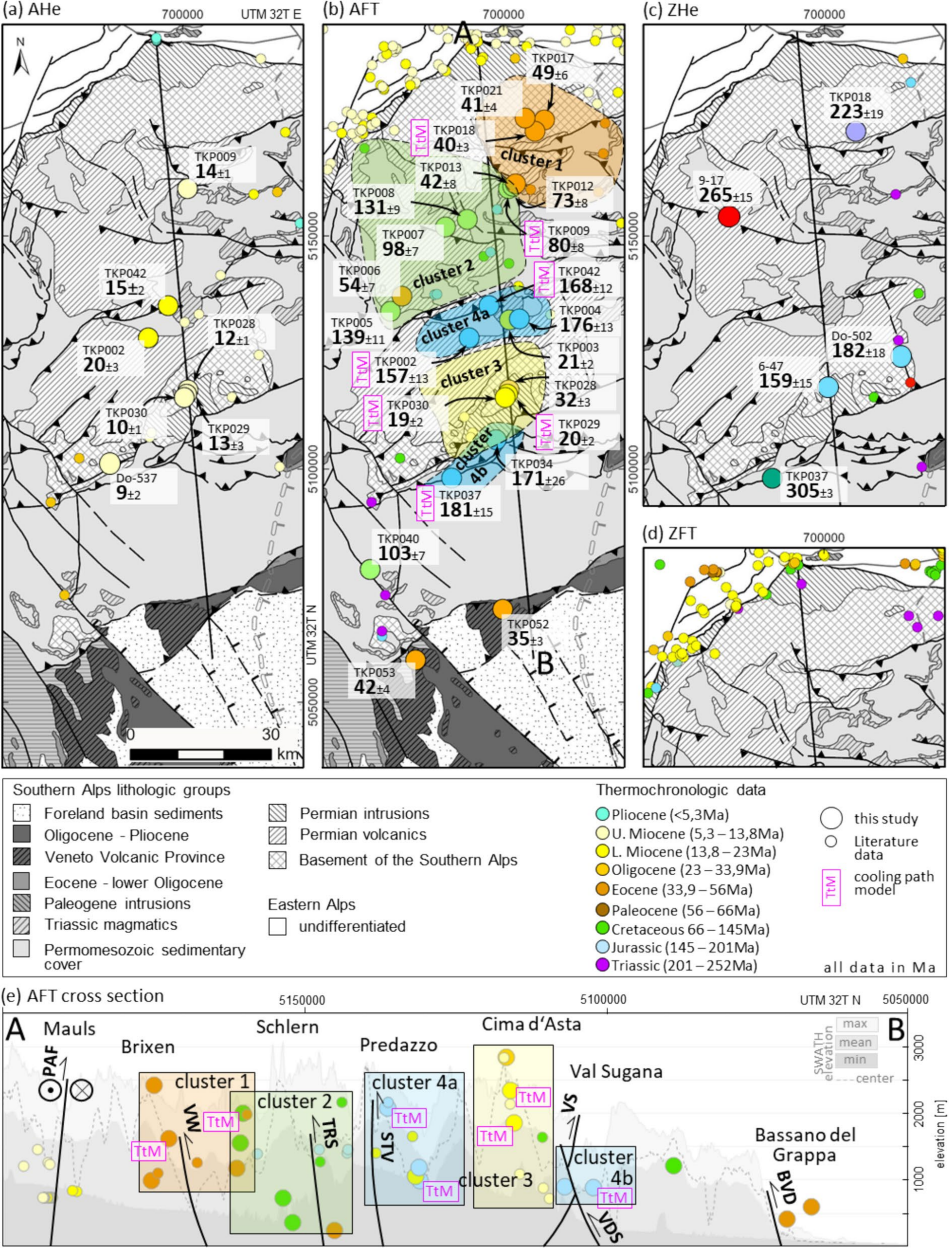
New and published **a** apatite (U-Th)/He data, **b** apatite fission-track data, **c** zircon (U-Th)/He data, and **d** published zircon fission-track data. Note, that apatite fission-track data cluster in four distinct domains (cluster 1–4). **e** Position of the AFT samples on a SWATH profile for a 25 km wide corridor along the cross section of Fig. 3. All data are color-coded by geological epochs in both map and cross section view. *AFT* apatite fission-track, *AHe* apatite (U-Th)/He, *BVD* Bassano-Valdobbiadene thrust, *PAF* Periadriatic fault system, *STV* Stava line, *TRS* Tiers fault, *VDS* Val di Sella thrust, *VS* Valsugana thrust, *VW* Villnöß/Val di Funes and Würzjoch/Passo del Erbe thrusts, *ZFT* zircon fission-track, *ZHe* zircon (U-Th)/He

Figure 4 shows the sampling locations, thermochronological (AFT, ZHe and AHe) data in map view, and a simplified and vertically exaggerated tectonic cross section containing the AFT data of this study. All samples are located on the Trento platform and were, therefore, covered by thinner sedimentary sequences compared to the adjacent basins (Sieberer et al., 2023; Verwater et al., 2021). The ages of sampled formations span from the Variscan basement to Middle Triassic sediments and (volcano-)clastics, with the exception of the southernmost two samples, which originate from the Cenozoic Venetian volcanic province. The projection onto the cross section was carried out parallel to the main ENE-WSW strike of the dominant Neoalpine tectonic structures. The admissible projection distance was carefully assessed for each individual sample based on the regional geology and tectonic structures to avoid projection related perturbations and overinterpretation. A swath profile covering a 25 km wide corridor is plotted as background elevation range indicator. Both the AFT map and the cross section allow for the discrimination of four spatially delimited clusters from new and published data, which are highlighted in both plots. It is worth noting that these clusters do not necessarily correspond to the tectonic blocks shown in Fig. 3.

## Discussion

**Fig. 5:**
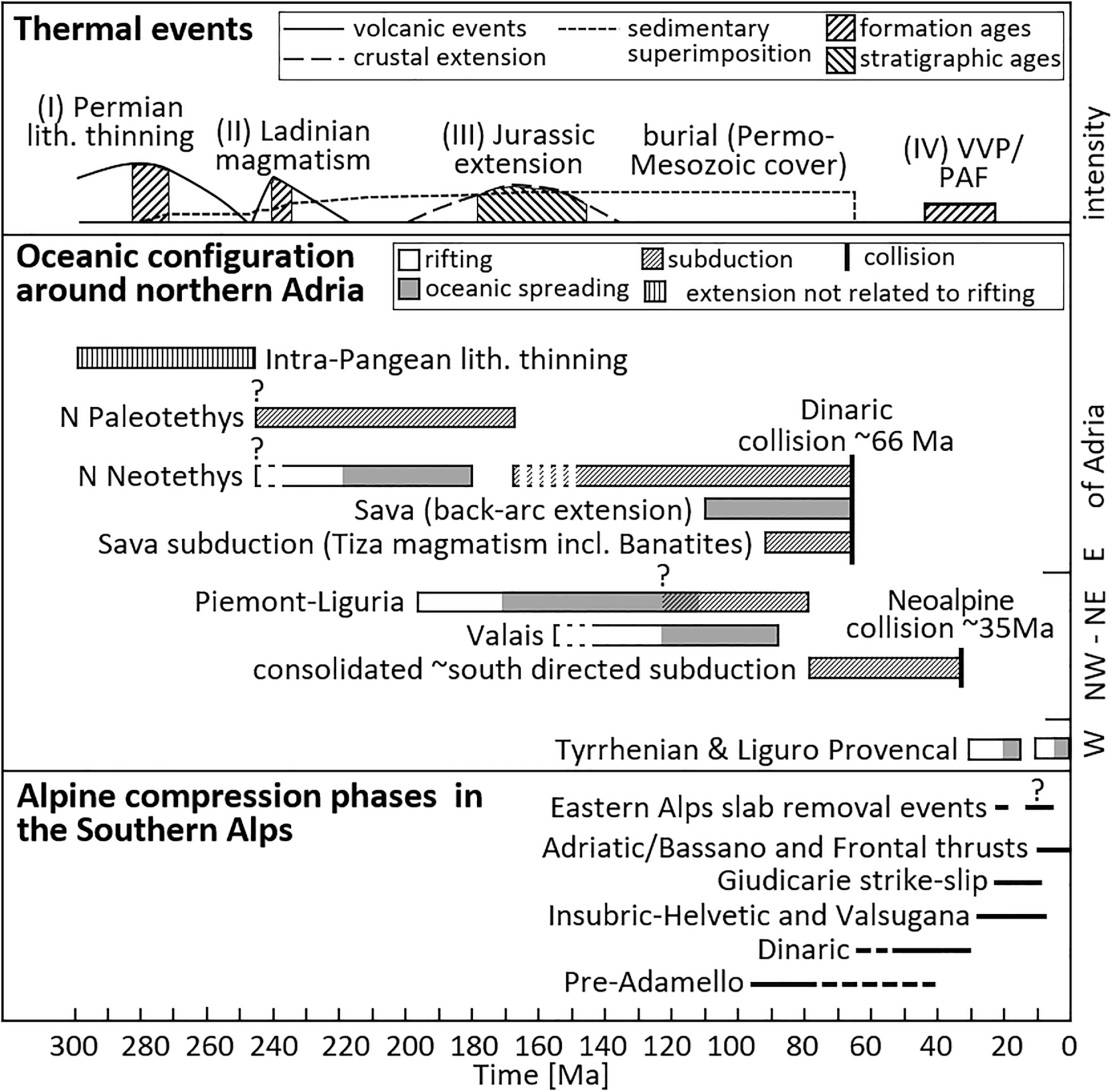
Compilation of regional processes related to subordinate tectonics for tectonic contextualization: post-Variscan thermal events (based on Carminati et al., 2010; Grobe et al., 2015; Mundil et al., 1996; Schuster & Stüwe, 2008; Storck et al., 2018), oceanic configuration around northern Adria (based on Agard & Handy, 2021; Handy et al., 2010; Handy et al., 2015; Le Breton et al., 2021; Pohl et al., 2018; Schmid et al., 2008; Speranza et al., 2002; Ustaszewski et al., 2009; van Hinsbergen et al., 2020), and Alpine compression in the eastern Southern Alps (based on Caputo et al., 2010; Castellarin et al., 2006b; McPhee & Handy, 2024; Picotti et al., 2022; Schmid, 2023). *VVP* Veneto volcanic province, *PAF* Periadriatic fault

Our new data constrain the thermal history for the Cenozoic orogenic phase of the ESA and the Mesozoic passive continental margin evolution and allows for a more refined treatment of existing thermal models. Figure 5 shows the thermal and tectonic framework for a proper contextualization of the data (see Sects. 1 and 2).

### The Mesozoic to Cenozoic AFT record

**Fig. 6:**
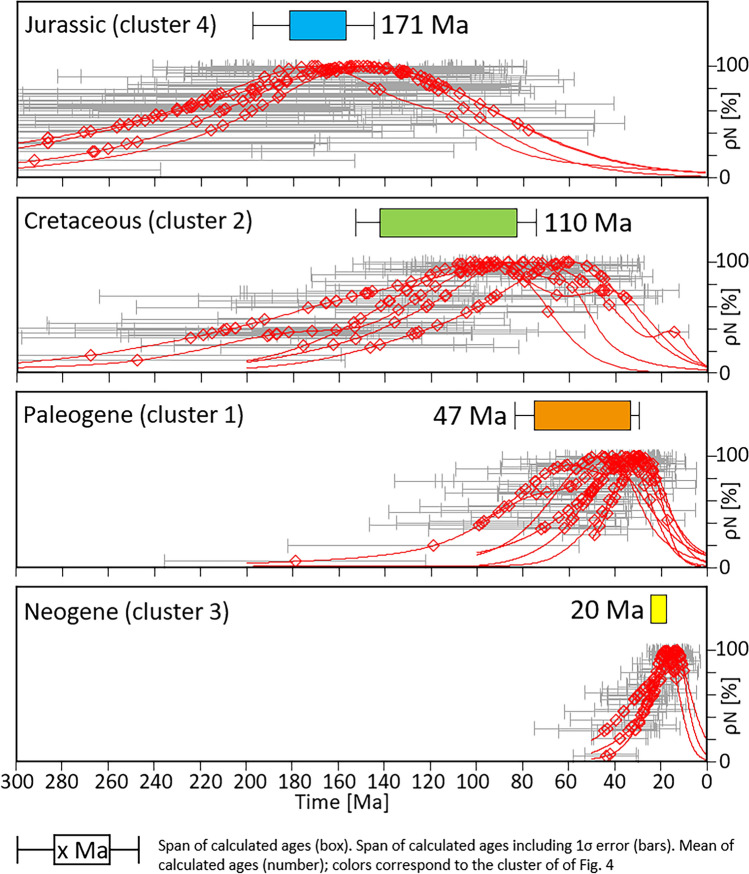
Apatite fission-track single-grain ages (red diamonds), single-grain age errors (black bars), and single-grain age distributions (red lines). Four groups of single-grain age distributions (Jurassic, Cretaceous, Paleogene, and Neogene) can be distinguished, corresponding to the clusters of Fig. 4. For each cluster the mean apparent AFT age (number), the range of calculated apparent ages (solid box, color corresponds to the clusters of Fig. 4), and the error span of the calculated apparent ages (bars) is applied. Distribution width increases roughly with the apparent age, older groups contain samples exhibiting wider distributions. The assignability of individual samples suggests exposure to similar conditions. Groups contain the following samples: Jurassic TKP002, TKP004, TKP037, TKP042; Cretaceous: TKP005, TKP007, TKP008, TKP009, TKP040; Paleogene: TKP006, TKP012, TKP013, TKP017, TKP018, TKP021, TKP028; Neogene: TKP003, TKP029, TKP030

AFT confined track length measurements are scarce for the majority of samples and the calculated ages alone do not allow for any reliable conclusions to be drawn about the associated thermal evolution. Consequently, the distribution of the single-grain ages is used to categorize the data and evaluate the calculated ages with respect to their thermal history (e.g., Fitzgerald & Malusà, 2019; Gallagher et al., 1998; Gleadow et al., 1986; O'Sullivan & Parrish, 1995). The data cluster into four groups (Fig. 6), each containing samples of similar apparent age and single-grain age distribution. These groups correspond to clusters 1–4 of Fig. 4 with the exception of TKP028.

Cluster 4 (Jurassic AFT data) is characterized by a wide, rather symmetric and unimodal single-grain age distribution. Apparent ages and single-grain age distribution maxima cluster during the drifting stage of the Piedmont-Liguria ocean. All samples pass the χ^2^-test and yield high P values of 49–89%. This strongly supports a limited variation in chemical composition, shared thermal history, and reset conditions for all single grains for these samples. This suggests that burial heating was insufficient to yield thermal equilibration following the Jurassic crustal extension. This is consistent with the findings of Zattin et al. (2006) who inferred cooling during Mesozoic passive margin sedimentation based on organic maturity data.

Cluster 2 (Cretaceous AFT data) displays the widest spread of apparent AFT ages, with a partially bimodal single-grain age distribution. Four out of five samples fail the χ^2^-test.

Five of seven samples assigned to cluster 1 (Paleogene AFT data) exhibit acceptable or high χ^2^ P values. The peak of the sample density functions is clustered in the late Eocene and the turning points are largely within the Paleocene. All samples were collected from the basement or the Permian part of the sedimentary cover. The majority of the data plot in the Eocene and correspond to the cooling phase of the Dinaric orogeny, with the exception of two data points (TKP012, TKP028).

Two (TKP029, TKP030) out of the three Neogene dates assigned to cluster 3 have been collected in the hanging wall of the Valsugana fault near Borgo. Both are very likely affected by tectonic activity during the formation of the ESA. The third sample (TKP003) was collected near Predazzo and is of uncertain tectonic origin. All three samples pass the χ^2^-test.

### Post-Variscan thermal events

The post-Variscan geodynamic evolution of the Southern Alps entails a polyphase thermal record of four major events and the sedimentary burial. At the same time, Variscan K–Ar and Ar–Ar data in white mica in the Brixen basement indicate a maximum post-Variscan regional temperature of < 350 °C (Hammerschmidt & Stöckhert, 1987).

**Fig. 7:**
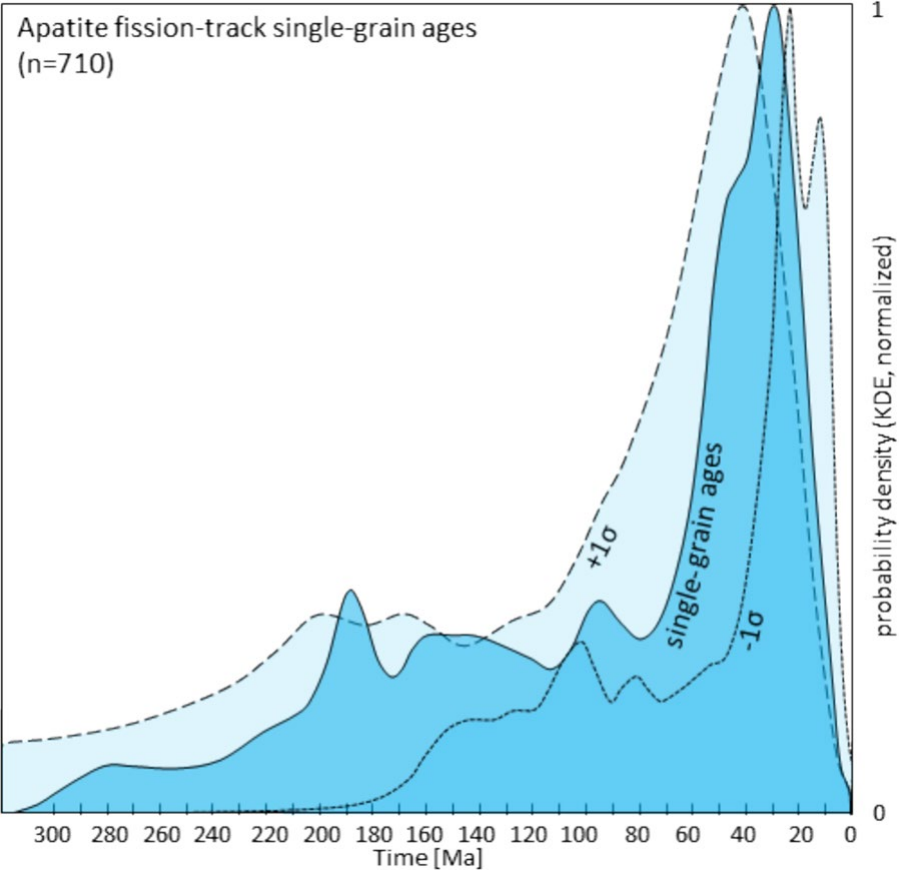
Probability density plot of apatite fission-track single-grain ages. Note that the vast majority of all AFT single-grain ages is younger than Middle Triassic. Note that the dashed lines do not represent the confidence interval for the probability density of the single-grain ages, but refer to the population of single-grain ages + 1σ and the population of single grain ages -1σ

- Permian thermal event (~ 290–260 Ma): Postdating the Variscan orogeny, the pre-Alpine basement was affected by Permian magmatism (e.g., Bonin, 1998; Bonin et al., 1993; Locchi et al., 2022; Schaltegger & Brack, 2007; Yuan et al., 2020). Schuster and Stüwe (2008) link the emplacement of Permian magmatites to a regional low-p/high-T metamorphic event. This event occurred well ahead of the Triassic opening of the Neotethys ocean and passive margin sedimentation. They propose that high-T peak metamorphism occurred at ~ 280 Ma with a substantial amount of thermal equilibration taking place before 240 Ma. Estimating the timing of the regional thermal overprint remains difficult as only a few data points recorded a Permian age: One ZHe date of 265 ± 10 Ma is derived from Valsugana thrust hanging wall basement rocks (sample Transalp-21, Eizenhöfer et al., 2021), and another date of 265 ± 15 Ma from the Athesian Volcanic Group south of Bozen (sample 9–17, this study). The late Carboniferous 305 ± 3 ZHe data point from the basement sample TKP037, located to the south of the Valsugana thrust, suggests that the regional thermal overprint may have been spatially limited.
- Ladinian magmatism (~ 242–238 Ma): The timing of Ladinian magmatic rock emplacement and the associated tectonic setting have been addressed in Sect. 2.1. Thermal history models of Tscherny et al. (2012) and Grobe et al. (2015), calibrated with vitrinite reflectance organic maturity data from the Permo-Mesozoic stratigraphic sequence, exhibit an early Triassic increase of temperature prior to Middle Triassic regional rapid subsidence of the Gröden/Val Gardena and Gadertal/Val Badia regions. A probability density plot of all new AFT single-grain ages reveals that only 4.6% of the data are older than the Ladinian (Fig. 7). Detrital ages and magmatic formation ages are overprinted throughout the study area. This indicates that temperatures well within the AFT PAZ were reached.
- Jurassic crustal thinning (~ 190–150 Ma): The Jurassic evolution of the geothermal gradient is linked to crustal extension caused by the opening of the Vardar Ocean in the (south)east and the Piedmont-Liguria Ocean in the (north)west of the Adriatic domain (see Sect. 2.1). A model proposed by Carminati et al. (2010) and based on organic matter maturity data from Fantoni and Scotti (2003) indicates a peak in heat flow for the western Trento platform (Non valley) of up to 100 mW/m^2^ at the basement top at 175 Ma. This is up to 40% above the Late Triassic level. The models of Tscherny et al. (2012) and Grobe et al. (2015) estimate a Jurassic temperature rise of 20–30% at ~ 175 Ma for the basement top, but suggest maximum temperatures of 160 °C. However, despite the presence of a peak in the single-grain age distribution associated with the Jurassic temperature increase (Fig. 7), we cannot observe a widespread reset of the AFT system in our new basement rock or Permian to Middle Triassic sedimentary rock samples.
- Permo-Mesozoic burial heating (~ 290–66 Ma): The various thermal models discussed concur that the maximum depth of Mesozoic and Cenozoic burial on the Trento platform cannot be held accountable for the maximum temperatures observed. The total thickness of the post-Variscan, Permian to Cenozoic volcano-sedimentary succession in the Southern Alps is approximated to be ~ 6 up to ~ 10 km in the Valcamonica (Berra & Carminati, 2010) for the Lombardian basin, ~ 3 to ~ 4.5 km for the Trento platform (Bertotti et al., 1993), ~ 8.5 km for the Belluno Basin, ~ 7 km for the Friuli platform and ~ 8.5 km for the Slovenian basin (Sieberer et al., 2023 and citations therein). Ring and Richter (1994a) estimate a sedimentary overburden for the Brixen area on the northern Trento platform of 4–5 km and an additional ~ 1 km of eroded basement, based on coalification of organic matter and conodont alteration index data. This is consistent with the findings of Tscherny et al. (2012) and Grobe et al. (2015), whose models aim for a maximum regional temperature of 115 to 140 °C at the top of the basement on the central Trento platform. The post-Variscan sediment thickness of the Venetian basin south of the Montello thrust is in the range of ~ 7.5 km, including ~ 3 km of Cenozoic synorogenic and syntectonic sediments (Doglioni, 1992b; Picotti et al., 2022). Their data are based on retro-deformation and thickness estimates from borehole and rock outcrop data.
- Alpine magmatism (~ 42–22 Ma): It is unlikely that the Paleogene magmatic events (Sect. 2.3) resulted in a regional thermal overprint. The emplacement of the Periadriatic intrusions was constrained to the narrow corridor of the Periadriatic fault system and the Adamello area (Castellarin et al., 2006a; Pomella et al., 2011). Subsequently, some of the plutonic bodies have undergone a vertical offset with respect to the Southern Alpine rocks as a consequence of transpressive faulting (e.g., Klotz et al., 2019; Pomella et al., 2011; Prosser, 1998). The occurrence of the Veneto Volcanic Province is restricted to the southwesternmost part of the ESA (Val d’Adige, Marostica Hills), partially affecting regions south of the frontal thrust system (Lessini Mountains, Berci Hills, Euganean Hills; Brombin et al., 2021; De Vecchi & Sedea, 1995; Macera et al., 2003). New AFT and AHe data derived from rocks of the Marostica Hills (TKP052, 35.3 ± 2.8 Ma) and the Lessini Mountains (TKP053, 42.3 ± 3.5 Ma) exhibit cooling ages corresponding to the time of their emplacement (Brombin et al., 2019, and references therein; Brombin et al., 2021; Savelli & Lipparini, 1979; Visonà et al., 2007a).

**Fig. 8:**
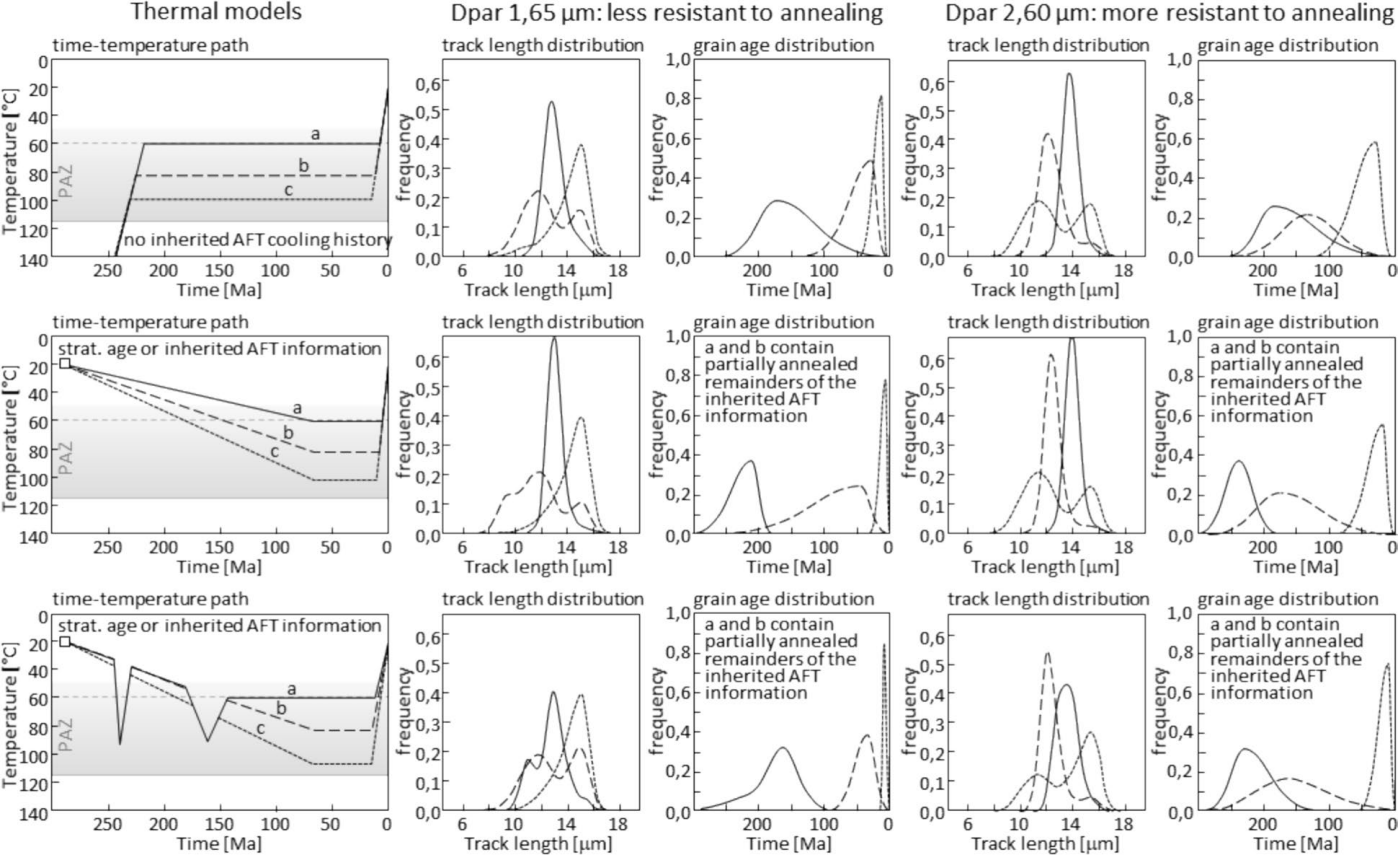
Schematic AFT track length and single-grain age distributions for simplified and expected time–temperature paths of the ESA for two different Dpar values representing the apatite composition and annealing ability. Track lengths and single-grain age distributions (central and oldest) are based on HeFTy forward models, O'Sullivan and Parrish (1995), Gleadow et al. (1986), Green et al. (1986), Gallagher et al. (1998), Malusà and Fitzgerald (2019b), and references therein. Note, that the spread of single-grain ages and track length variability is greatest at intermediate temperatures within the PAZ (80–100 °C), which goes along with the lowest sensitivity but highest spread of possible paths. Abbreviations: Dpar arithmetic mean of AFT etch pit lengths parallel to the crystallographic c-axis (Donelick, 1993)

The presence of multiple thermal events and the absence of a high temperature overprint can have the effect of misleadingly influencing the geological interpretation of low-temperature thermochronological data. Accordingly, we employed thermal forward models to predict perturbation patterns and hypothesize end-member states of ESA cooling paths. Figure 8 provides a schematic illustration of the effect that inherited age data, temperature, residence time, and multiple thermal events can have on single-grain age and confined track length distribution of AFT data based on synthetic cooling paths. The schematic single-grain age distribution provides some guidelines for the thermal history of samples without sufficient confined track length measurements for a time–temperature modelling. However, a reliable cooling path cannot be derived from this.

Based on the dispersion of single-grain ages in individual samples (Fig. 6), and keeping in mind the boundary conditions of the thermal events during burial and cooling (Fig. 8), we assign AFT data that are not related to the Cenozoic tectonic events to the presence of a widespread, exhumed, long-lived Triassic to Cretaceous AFT PAZ. This assumption can explain the wide range of single-grain ages yielding Mesozoic AFT data, and allows for a shift or partial reset of the AFT system due to burial without exceeding the closure temperature for a full reset.

### Thermotectonic history restored along the N-S Trento platform transect

Cooling ages constrain the thermal history of a rock and are bound to the closure temperature *Tc* of a specific thermochronological system, which varies according to the cooling rate (e.g., Jäger, 1967; Malusà & Fitzgerald, 2019b; Wagner et al., 1977). This concept requires cooling to occur monotonically from above *Tc* to low temperatures (Dodson, 1973). In order to apply this concept to a fold-and-thrust belt, a sufficient amount of initial overburden is required to ensure a geothermal reset prior to the exhumation. A major consequence of the thermal history discussed in Sect. 6.2 is that conditions in the ESA rarely reset the AFT system. Affiliated data must therefore be considered apparent ages. However, thermal history modelling provides a means of constraining cooling paths. Our data enabled time–temperature models to be created for all the tectonic blocks involved in the exhumation history of the ESA along the Trento Platform cross section (Fig. 3).

#### The ESA indenting tip: slow Dinaric and long-term Neoalpine cooling

We refer to the ESA Indenting Tip as the northernmost tectonic block of the ESA, located between the Periadriatic fault system and the Villnöß and Würzjoch thrusts. These represent the northernmost backthrusts of the Valsugana thrust system (Castellarin et al., 1998), and their geometric westward extension (Fig. 3).

*The post-Variscan thermochronological record:* Permian Rb–Sr biotite data from Borsi et al. (1978) report fast, post-emplacement cooling below ~ 300 °C for the Brixen granite intrusion, representing the present-day, northernmost lithology of the ESA (Fig. 1). ZFT age-elevation analyses conducted on basement samples south of Bruneck/Brunico (Most, 2003) suggest fast post-Ladinian cooling, post-dating the Middle Triassic thermal event (Sect. 6.2). Cretaceous ZFT data presented by Stöckli (1995), Klotz et al. (2019), and Pomella et al. (2012) from the northwestern Brixen granite intrusion, along with the corresponding wide single-grain age distributions of their samples argue against an interpretation as cooling ages. In combination with the total absence of tectonically driven Cretaceous cooling ages in AFT and AHe data this does not acknowledge Eoalpine exhumation in the ESA. Visonà et al. (2022) propose the possibility of an Oligocene local thermal overprint caused by a Periadriatic pluton beneath the Brixen granite intrusion based on Ar–Ar data from metasomatic K-feldspar. However, the authors limit the affected area to the west of the study's sampling corridor.

**Fig. 9:**
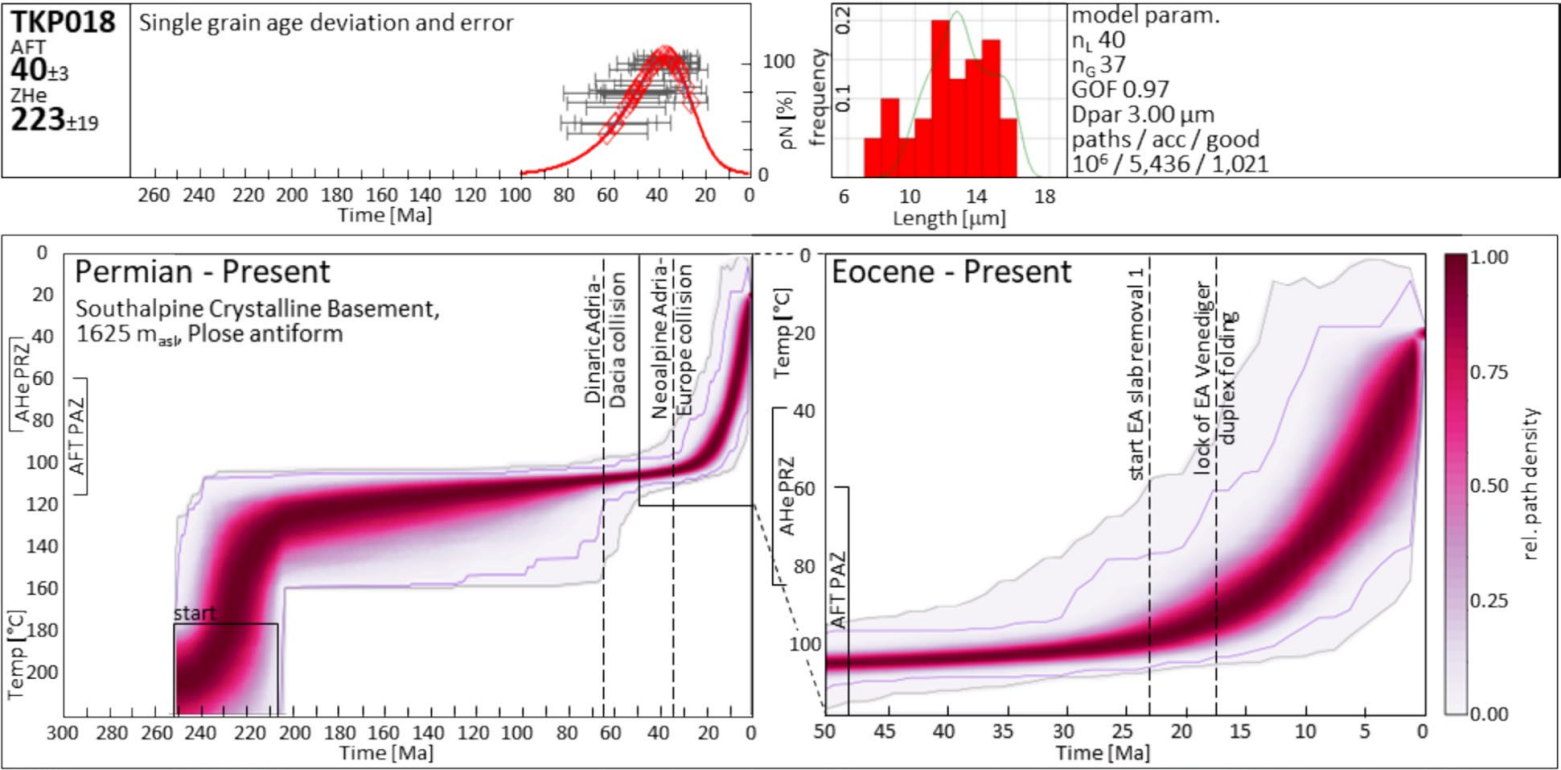
TKP018 single-grain ages, track length distribution, and time–temperature path. Model starting conditions are based on a ZFT date of the basement sample S134 from Pomella et al. (2012) which matches the age range of ZFT basement samples from Most (2003). The heat map represents a visualized statistic of modelled t-T path occurrences with the purple and grey envelopes limiting the ranges of good and acceptable paths, respectively. *acc* acceptable, *AHe* apatite (U-Th)/He, *AFT* apatite fission-track, *Dpar* arithmetic mean of AFT etch pit lengths parallel to the crystallographic c-axis (Donelick, 1993), *EA* Eastern Alps, *GOF* goodness of fit, *n*_*L*_ number of measured confined tracks, *n*_*G*_ number of analyzed crystals, *PAZ* partial annealing zone, *PRZ* partial retention zone, *rel* relative, *ZHe* zircon (U-Th)/He

*Slow Dinaric cooling:* The ESA Indenting Tip partially overlaps with the Eocene age cluster of AFT data east of Brixen (cluster 1 in Fig. 4). The occurrence of this cluster spatially coincides with the hanging wall of the assumed Dinaric thrust front (Doglioni, 1987), and it can be clearly distinguished from the dominant Cretaceous AFT cluster to the southwest. A time–temperature model for the Plose massif east of Brixen reveals converging cooling paths within the upper limit of the AFT PAZ during the Eocene (TKP018, Fig. 9). Structural field data from the Brixen basement (Baggio et al., 1969) indicate that the summit of the Plose massif is the hinge zone of a mountain-scale antiform with a steep northwest-southeast trending axial plane, consistent with the Dinaric top ~ southwest shortening direction.

The spatial pattern of the thermochronological data, when considered alongside the aforementioned structural observations, is most likely connected to the presence of Dinaric structures. These include (i) the Plose antiform, and (ii) the northwest-southeast trending St. Ulrich-Klausen thrust (nomen novum, Figs. 1 and 3) which is necessary to decouple the cluster of Eocene cooling ages to the southwest. Thrust-terrain intersection and the general trend of the mapped Dinaric structures suggest a geometric link between the St. Ulrich-Klausen thrust and the Digonera thrust. The latter is exposed in the Buchensteintal/Valle di Livinallongo and has been extensively described by Doglioni (1992a). However, the absence of surface expressions of this connection may indicate a blind fault-and-fold geometry.

**Fig. 10:**
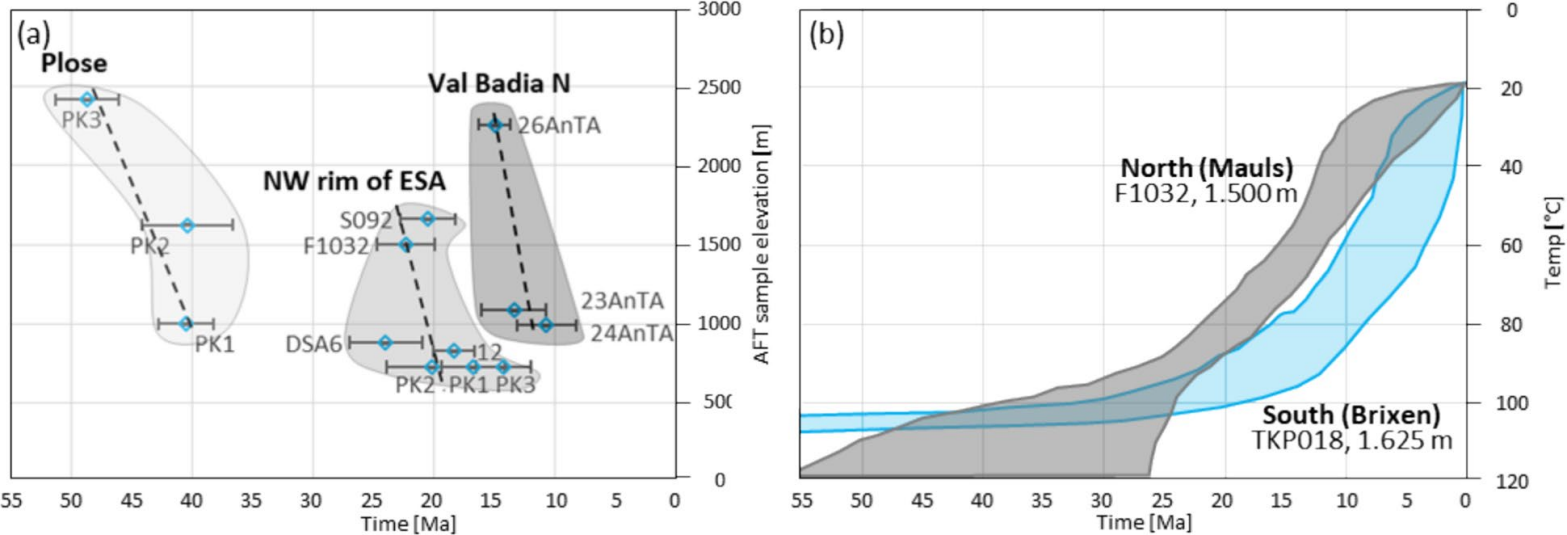
**a** The regional age-elevation correlation from the ESA Indenting tip carves out the effect of focused tectonic uplift at the time of Eocene Dinaric slow exhumation of the Plose antiform, Early Miocene Neoalpine exhumation at the NW rim of the ESA, Middle Miocene Neoalpine exhumation in the northern Val Badia. **b** North-side-up tilting recorded by the cooling paths of F1032 and TKP018 (good paths, rel. path density 0.5)

Dinaric thrusting in the northern ESA requires basement involvement, at least for the Brixen and Comelico areas (Fig. 1). This is particularly noteworthy since Dinaric nappe stacking and thrusting within the ESA are considered to be thin-skinned in most of the literature (e.g., Brandner & Gruber, 2019b; Doglioni, 1987; Massari et al., 1986). The assumption is consistent with the general thinning of the evaporite bearing Bellerophon Fm., which acts as a preferred detachment in the ESA, from the Comelico-Cadore basin towards the west (Cassinis et al., 1997). AFT age-elevation correlation of TKP017, TKP018 and TKP021 from the Brixen basement of the Plose massif allows an estimate for the apparent exhumation rate at the northwestern Eocene Dinaric frontal thrust of ~ 0.17 km/Myr (Fig. 10, age range 48.7–40.5 Ma, elevation range 2,419–998 m).

*Long-term Neoalpine cooling:* An apparent exhumation rate of 0.4 km/Myr, attributed to Neoalpine tectonics can be inferred from the age-elevation correlation of data presented by Most (2003), based on the samples 23AnTA, 24AnTA, and 26AnTA from Gröden (age range 15.0–10.7 Ma, elevation range 2.260–990 m). The coexistence of the Eocene Plose record and the Middle Miocene Gröden record at an equivalent elevation range is most likely attributed to the ramp and flat geometry of the Dinaric thrust belt and its northern termination. There, Neoalpine thrusting on the Villnöß and Würzjoch thrusts decreases towards the west (Fig. 1), and the focused Neoalpine backthrusting ceases in the Eisacktal/Valle Isarco.

Neoalpine exhumation of the northernmost ESA Indenting Tip has been constrained by Pomella et al. (2012) based on modelled time–temperature paths on their sample F1032 from the Brixen granite intrusion. Even though exhumation for the northernmost ESA prior to the Valsugana phase can be inferred from this model, the timing of its onset is uncertain. Our TKP018 time–temperature model (Fig. 9) confirms a pre-14 Ma exhumation for the northernmost ESA and constrains this onset of fast exhumation to the late Oligocene. Thus, it takes place even before the onset of Giudicarie thrusting and the Adriatic Indenter dissection. A comparison of the FT1032 and TKP018 cooling paths indicates a north-side-up tilting from the Chattian onward, due to the initial enhanced cooling in the north, with the southernmost cooling path lagging behind (Fig. 10). This tilting is also obvious from AFT map view data, where Oligocene to Miocene data (25–10 Ma) are clustered to the north of the ESA Indenting Tip, but do not occur south of Brixen (Fig. 4). It is also consistent with major age populations in detrital fission-track samples from the Piave river and the Venetian basin between 24 and 17 Ma (Monegato et al., 2010). The process responsible for this observation remains uncertain. The post-collisional thrusting of the indenting Adriatic leading edge onto the sub-Tauern ramp (TRANSALP Working Group, 2002, correction published 2006), the formation of an accretionary crustal bulge (Kummerow, 2003), and an early crustal wedging of accreted European crust underneath the ESA, similar to the model of McPhee and Handy (2024), are reasonable mechanisms. It is noteworthy that the northern tip of the ESA rests on a zone of thickened high seismic velocity crust (Jozi Najafabadi et al., 2023) (Fig. 2). The cessation of tilting in the Middle Miocene does not support a westward extension of the Middle to Late Miocene wedging of European upper crust beneath the ESA, as proposed by McPhee and Handy (2024) for the TRANSALP transect. The post-Valsugana phase decrease in the cooling rate of sample F1032 (Pomella et al., 2012) and the contemporaneous increase in the cooling rate of sample TKP018 (Fig. 10) suggest a deep-seated flat-ramp transition of the Bassano-Valdobbiadene thrust system below the ESA Indenting Tip.

#### Central Dolomites Pop-up: backthrusting, piggyback transport and the Miocene Predazzo thermal anomaly

The Central Dolomites Pop-up adjoins the ESA Indenting Tip to the south (Fig. 3). A tectonic separation towards the north is provided by the Villnöß and Würzjoch thrusts, the northernmost backthrusts within the ESA. The southern limit is confined by the frontal ramp kink of the Valsugana thrust, south of Predazzo, and excludes the Valsugana frontal ramp. The cross section (Fig. 3) shows a pronounced flat section of the Valsugana thrust underneath the southern Central Dolomites Pop-up.

*Jurassic and Cretaceous thermal evolution:* The northern Central Dolomites Pop-up hosts the southern part of the Eocene AFT cluster (cluster 1, Fig. 4), as discussed in Sect. 6.3.1, as well as clusters of mixed Cretaceous and Jurassic AFT cooling data (cluster 2, Fig. 4), and of Jurassic AFT data (cluster 4a, Fig. 4). Single-grain age distributions in samples of Jurassic apparent age range from the Permian (though with a large error) to the Late Cretaceous and are unimodal and symmetric (majority 250–100 Ma). Samples of Cretaceous apparent age, contain single-grain ages ranging from the latest Permian to the Miocene (majority 225–50 Ma) and comprise uni- and bimodal distributions (Fig. 6).

**Fig. 11:**
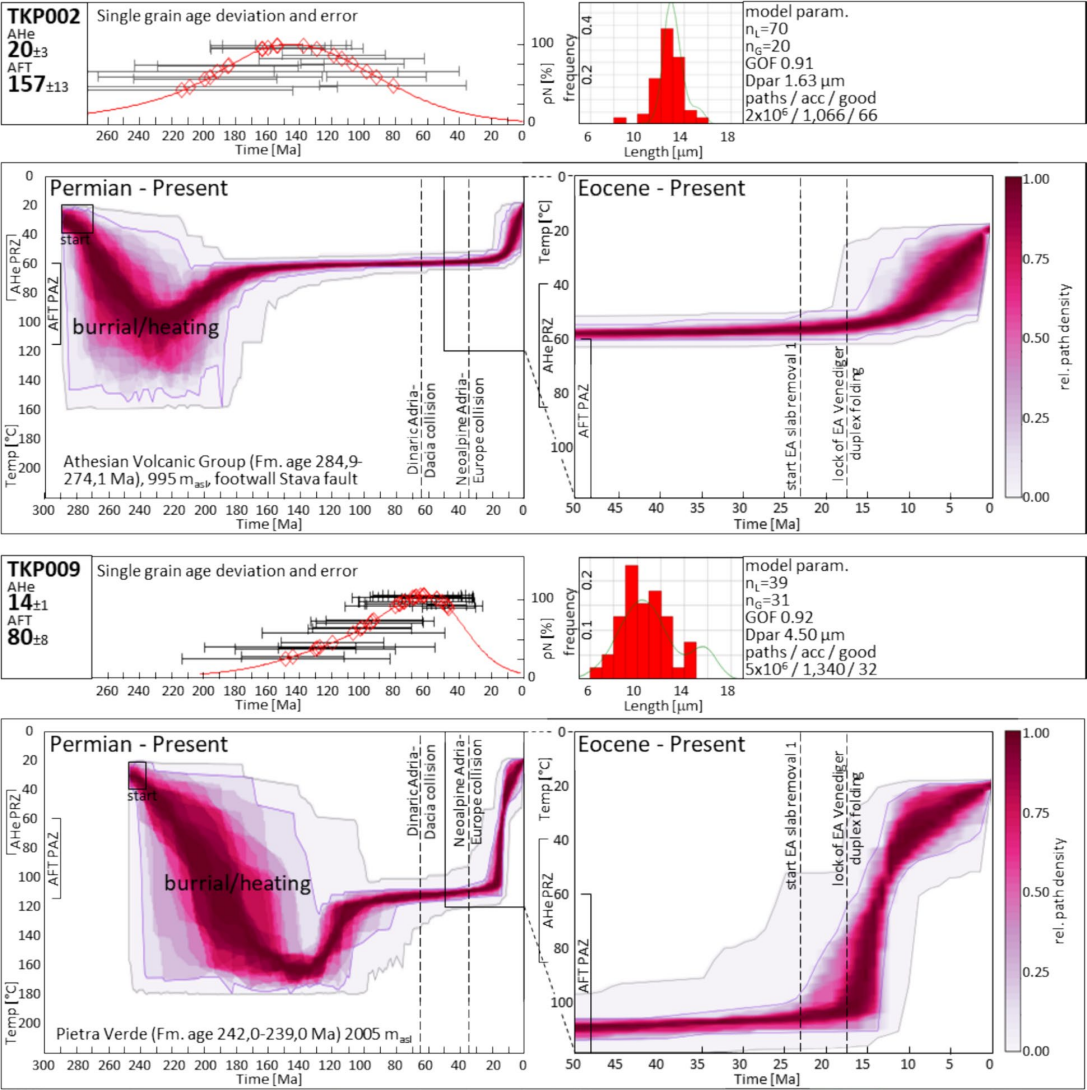
Single-grain ages, track length distribution, and time–temperature paths from samples in the Central Dolomites Pop-up TKP002 and TKP009. Starting condition for all samples are the respective deposition ages of the sedimentary rocks. Therefore, subsequent burial or heating is required. The heat map represents a visualized statistic of modelled t-T path occurrences with the purple and grey envelopes limiting the ranges of good and acceptable paths, respectively. *acc* acceptable, *AHe* apatite (U-Th)/He, *AFT* apatite fission-track, Dpar arithmetic mean of AFT etch pit lengths parallel to the crystallographic c-axis (Donelick, 1993), *EA* Eastern Alps, *Fm.* formation, *GOF* goodness of fit, *n*_*L*_ number of measured confined tracks, *n*_*G*_ number of analyzed crystals, *PAZ* partial annealing zone, *PR*Z partial retention zone, *rel* relative

Missing post-rift tectonics combined with a wide range of single-grain ages resemble the thermal response of a passive continental margin to thermal relaxation as proposed by McKenzie (1978). This assumption is visible through the distinct post-rift thermal steady-state of cooling paths for the Trento platform. The cooling paths of TKP009 (Seiseralm/Alpe di Siusi, Buchenstein Fm., upper AFT PAZ ~ 110–20 Ma) and TKP002 (Predazzo, Athesian Volcanic Group, lower AFT PAZ ~ 180–20 Ma) are representative for this observation (Fig. 11). Mesozoic thermal steady-state can additionally be observed in our samples TKP030, TKP037, TKP008, TKP018 and in the time–temperature models of Zattin et al. (2003), their sample G1149, Zattin et al. (2006), their sample DO1, and Emmerich et al. (2005), their samples AE11, AE12, and AE18. TKP009 records the Jurassic thermal event and enters a phase of minor cooling after the thermal relaxation in the Cretaceous at a temperature of ~ 110 °C in the uppermost AFT PAZ. The cooling path of TKP002 distinctively displays only the Ladinian thermal event and remained in the lowermost AFT PAZ after slow thermal relaxation. Given these observed steady-state temperature ranges, the single-grain age distribution and the Mesozoic thermal pulses, we link the Jurassic and Cretaceous AFT data to the existence of an exhumed AFT PAZ on the passive Adriatic continental margin. This includes the Permian to Ladinian stratigraphy and partly the basement top of the ESA. Our interpretation is consistent with the concept proposed by Fitzgerald and Malusà (2019), who link the conservation of the slightly inclined age-elevation signature of exhumed PAZs to a subsequent phase of rapid cooling.

*Neoalpine Pop-up cooling:* The cooling path of sample TKP009 (Fig. 11) exhibits a period of fast cooling that commenced at ~ 20 Ma and persisted until ~ 10 Ma. This fast cooling event indicates a significant tectonic pulse. The most probable causes for this are activity along the steeply northward dipping, deep-seated Valsugana ramp, or a contemporaneous activity along both, of the Valsugana ramp and associated backthrusts. In contrast, the post ~ 10 Ma Adriatic/Bassano phase cooling is weak in comparison.

The strong early to Middle Miocene pulse fades southwards. Time–temperature modeling of TKP008 (Schlern/Sciliar region, east of Bozen) reveals moderate cooling from ~ 10 Ma onwards (Appendix, Fig. AP 3.1). TKP002 was sampled southwest of Predazzo, just north of the pronounced kink in bedding and foliation, which reflects the onset of the Valsugana thrust frontal ramp (Fig. 3). Major Neoalpine cooling as well initiated only after ~ 10 Ma.

Along the southern Central Dolomites Pop-up, time–temperature models suggest only a minor effect of the Valsugana phase, consistent with a very low angle or subhorizontal upper crustal Valsugana thrust. A regional, moderate response to Adriatic/Bassano phase cooling is evident in all samples, indicating piggyback transport along a moderately inclined Bassano-Valdobbiadene thrust ramp.

**Fig. 12:**
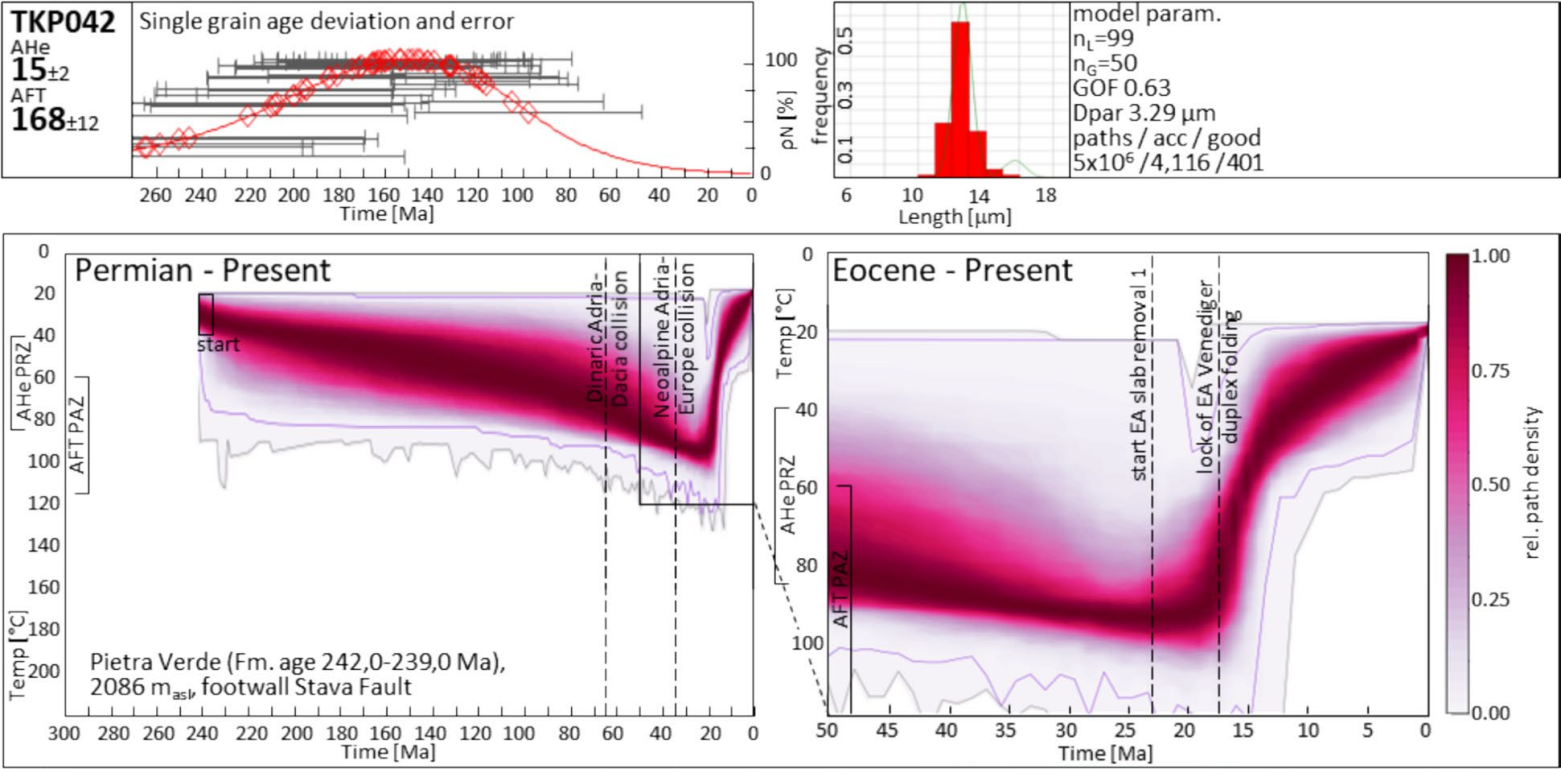
Single-grain ages, track length distribution, and time–temperature paths from sample TKP042 in the Central Dolomites Pop-up. Starting condition is the respective deposition age of the sedimentary rocks. Therefore, subsequent burial or heating is required. The heat map represents a visualized statistic of modelled t-T path occurrences with the purple and grey envelopes limiting the ranges of good and acceptable paths, respectively. *acc* acceptable, *AHe* apatite (U-Th)/He, *AFT* apatite fission-track, Dpar arithmetic mean of AFT etch pit lengths parallel to the crystallographic c-axis (Donelick, 1993), *EA* Eastern Alps, *Fm.* formation, *GOF* goodness of fit, *n*_*L*_ number of measured confined tracks, *n*_*G*_ number of analyzed crystals, *PAZ* partial annealing zone, *PRZ* partial retention zone, *rel* relative

*Miocene Predazzo thermal anomaly:* A phenomenon that requires elucidation is the spatial interfingering of Jurassic (TKP002, TKP004, TKP0042) and Miocene (TKP003; DO9 of Zattin et al., 2006) AFT data in the Predazzo region, in the absence of separating tectonic elements. The intensely fractured rocks in the vicinity of Predazzo have undergone synmagmatic Triassic dissection (Abbas et al., 2018; Carminati & Doglioni, 2022) as well as Neogene fracturing which is geometrically associated with the southwest-northeast striking Pinè and Fersina faults (Fig. 1), both being backthrusts to the Valsugana thrust system (Bigi et al., 1990; Selli, 1998). The time–temperature model of TKP042 (Fig. 12) from Passo Feudo northwest of Predazzo, indicates the existence of a Miocene thermal perturbation and a reset of the AHe system, though without a visible effect on the AFT single-grain ages. The simultaneous presence of (i) unperturbed Jurassic AFT data, (ii) a Jurassic AFT date exhibiting thermal perturbation in the time–temperature model, and (iii) Miocene AFT data, can potentially be explained by assuming a local thermal event focused only in the immediate vicinity of pre-existing, hydrothermal pathways.

#### Valsugana frontal ramp: long lasting Neoalpine thrusting

The tectonic units between Predazzo and Borgo Valsugana represent the frontal ramp fold in the hanging wall of the Valsugana thrust (Fig. 3) where crystalline basement and Permian intrusive rocks (Fig. 1) are exposed. Estimates by Dunkl et al. (1996) suggest 3–4 km Neoalpine erosion of overlying stratigraphy, based on Late Miocene AFT data.

The AFT data exhibit a west to east younging from Triassic and Cretaceous apparent ages at the western Valsugana valley head (which we attribute to the Mesozoic AFT PAZ as discussed earlier) to Miocene cooling ages near Borgo Valsugana and the Cima d’Asta massif (this study, Heberer et al., 2016), without a distinct tectonic separation. Yet, AHe data from the Caldonazzo area (western Valsugana valley), provided by the same authors, show middle to late Oligocene ages, whereas new and published data from the Cima d’Asta massif are of Late Miocene age, though at a higher elevation.

**Fig. 13:**
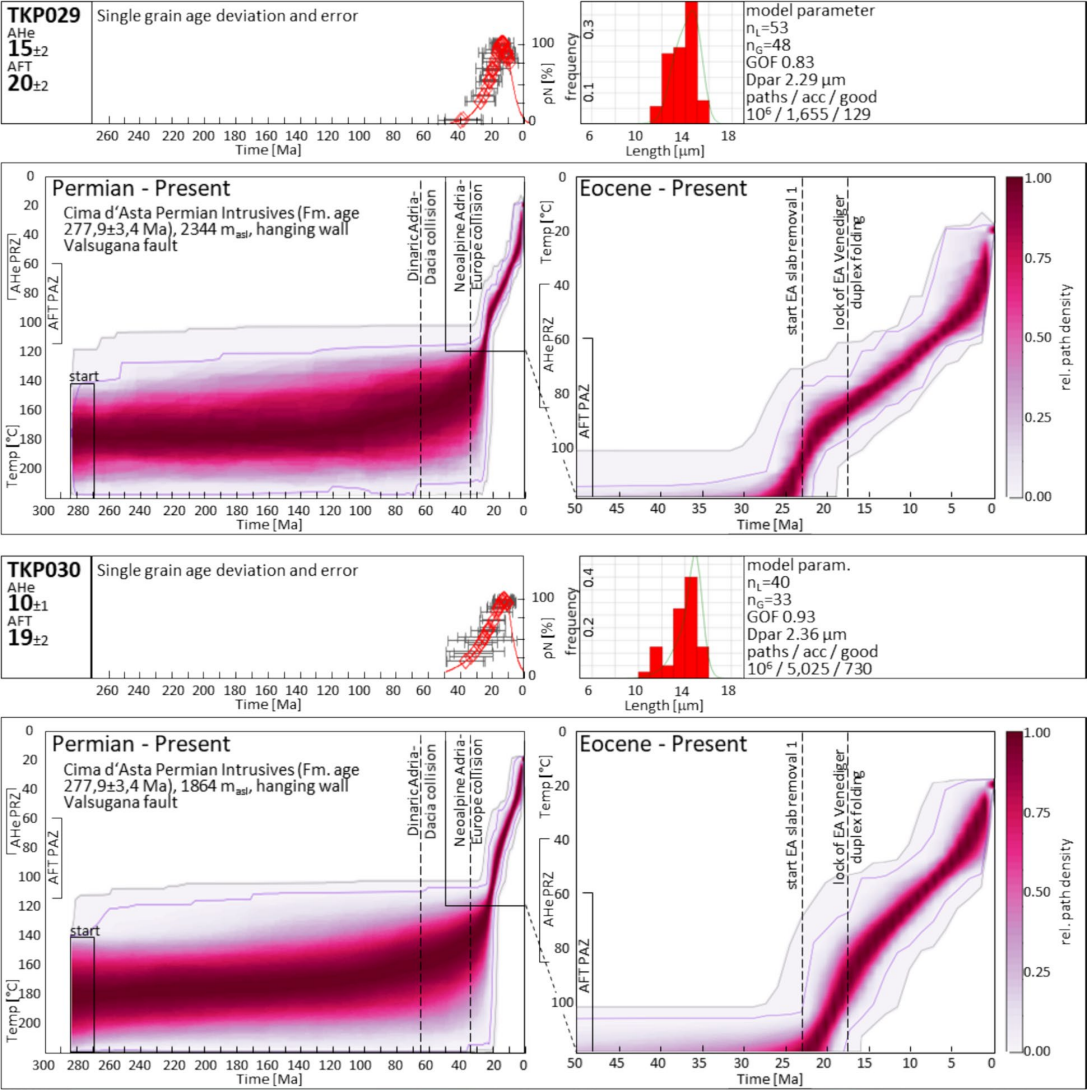
Single-grain ages, track length distribution, and time–temperature paths from Valsugana frontal ramp samples TKP029 and TKP030. Starting conditions for all samples are the respective deposition age of the sedimentary rocks. Note, that cooling starts earlier than the previously anticipated onset of Valsugana phase thrusting. The heat map represents a visualized statistic of modelled t-T path occurrences with the purple and grey envelopes limiting the ranges of good and acceptable paths, respectively. *acc* acceptable, *AHe* apatite (U-Th)/He, *AFT* apatite fission-track, Dpar arithmetic mean of AFT etch pit lengths parallel to the crystallographic c-axis (Donelick, 1993), *EA* Eastern Alps, *Fm.* formation, *GOF* goodness of fit, *n*_*L*_ number of measured confined tracks, *n*_*G*_ number of analyzed crystals, *PAZ* partial annealing zone, *PRZ* partial retention zone, *rel* relative

These observations are consistent with the late Oligocene onset of cooling in the TKP029 and TKP030 time–temperature models (Fig. 13), as well as with the Oligocene AFT apparent age of TKP028. Altogether, this strongly suggests the occurrence of cooling prior to the deposition of the youngest undeformed footwall sediments of the Valsugana thrust in the Langhian (Castellarin et al., 1987), which so far has been a major constraint on the earliest onset of Valsugana thrust activity. Tectonically, the data suggest a strong gradient of increasing exhumation in the hanging wall of the Valsugana thrust from its western termination to the zone of intense stacking east of Borgo Valsugana (Fig. 1), which includes the Valsugana, Belluno, Moline and Tezze thrusts (Curzi et al., 2024; Doglioni, 1992b; Zuccari et al., 2022). For the latter region, the clustering of recently published U–Pb data from tectonic carbonates and K–Ar data indicating fault gouge formation (Curzi et al., 2024) provide further evidence for intense tectonic activity from the Oligocene to the Late Miocene. However, we do not agree with their assumption of persistent Late Cretaceous to Late Miocene slow exhumation at the Valsugana thrust system, as our findings do not support the consistent interpretation of thermochronological data from the ESA as cooling ages (Sect. 6.1).

Our Oligocene to Early Miocene thermal evolution models (Fig. 13) correspond in time to (i) the decoupling of the Dolomites and Insubric Indenters due to incipient Giudicarie fault activity, and (ii) the evolution of flexural bending in the Southern Alps. The latter is associated with forebulge evolution, progressive curvature and, hence, increasing deviatoric stress, and is pronounced in the western and central Southern Alps (Bertotti et al., 1998). For the ESA, Picotti et al. (2022) suggest a similar retro-wedge flexural evolution, though less pronounced. This is due to the increased resistance of the strengthened Trento platform to deformation and the increasing distance between the Southern Alps and the Apennines towards the east (e.g., Bertotti et al., 1998; Caputo et al., 2010; Milli et al., 2024). An Oligocene forebulge formation initiating south of the Athesian Volcanic Group would allow pre-Langhian non-fault-bound exhumation that facilitated coeval fault activity at the Valsugana thrust system.

**Fig. 14:**
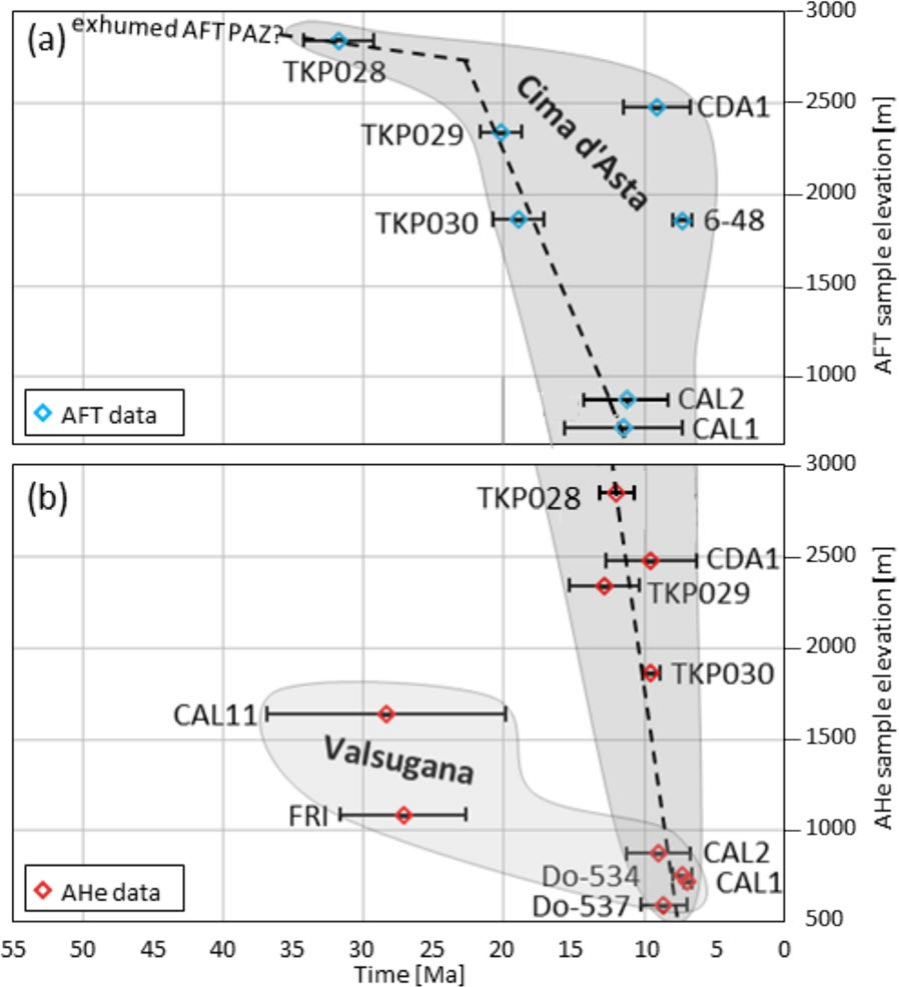
Regional age-elevation correlation from the Valsugana thrust frontal ramp based on apatite fission-track data (**a**) and apatite (U-Th)/He data (**b**). The Cima d’Asta elevation section shows fast apparent Neoalpine exhumation commencing in the Early Miocene. Towards the western part of the Valsugana valley, data are limited but suggest slower exhumation. Apparent exhumation rates (inclination of dashed lines) yield ~ 0.21 km/Myr based on apatite fission-track data and ~ 0.5 km/Myr based on apatite (U-Th)/He data. *AFT* apatite fission-track, *AHe* apatite (U-Th)/He

The age-elevation compilation (Fig. 14) restricts apparent exhumation rates to ~ 0.21 km/Myr in the Early and Middle Miocene (~ 22–11 Ma) and ~ 0.5 km/Myr in the Late Miocene (~ 11–9 Ma). In this context it is necessary to discuss the Tortonian AFT data from the Cima d’Asta massif presented by Dunkl et al. (1996), their sample 6–48, and Heberer et al. (2016), their sample CDA1, which clearly diverge from the Burdigalian data of TKP028 and TKP029, though sampled at the same altitude (~ 1,800–2,300 m), and the Rupelian data of our uppermost sample TKP028. In the light of Serravallian to Tortonian AHe data (this study; Heberer et al., 2016) from the same elevation, which are older than the reported AFT data, and Tortonian to early Messinian AHe data from ~ 700–900 m elevation, it is more reasonable to assume that the AFT data should be older than Serravallian. We, therefore, consider the data from this study to be more significant.

Based on the aforementioned data, we estimate a minimum of eroded overlying units for the Cima d’Asta summit to be in the range of 2.5–3.2 km, with a maximum thickness of 3.5–4.5 km, considering a geothermal gradient of either 30 °C/km (e.g., Malusà & Fitzgerald, 2019a) or a transient, tectonically induced ~ 37 °C/km as reported in the thermal model by Eizenhöfer et al. (2023). This requires a certain amount of eroded basement, as indicated by the exposure of the Cima d’Asta intrusive complex. However, thermobarometric analyses reveal a very shallow intrusion depth ranging from ~ 1 km (0,5 kbar; D'Amico & Franceschini, 1985) to 3–7 km (0,9–2,1 kbar; Acquafredda et al., 1997). Geological maps show the primary vicinity of the intrusion boundary and the basement top in the hanging wall of the Valsugana thrust (Fig. 1).

#### Bassano block: formation ages and reduced Permo-Mesozoic thermal perturbation

With the term Bassano block, we refer to the tectonic unit in the hanging wall of the Bassano-Valdobbiadene thrust and in the footwall of the Valsugana thrust (Fig. 3). Its western part comprises the Val di Sella backthrust, which locally offsets the Valsugana thrust (Selli, 1998).

**Fig. 15:**
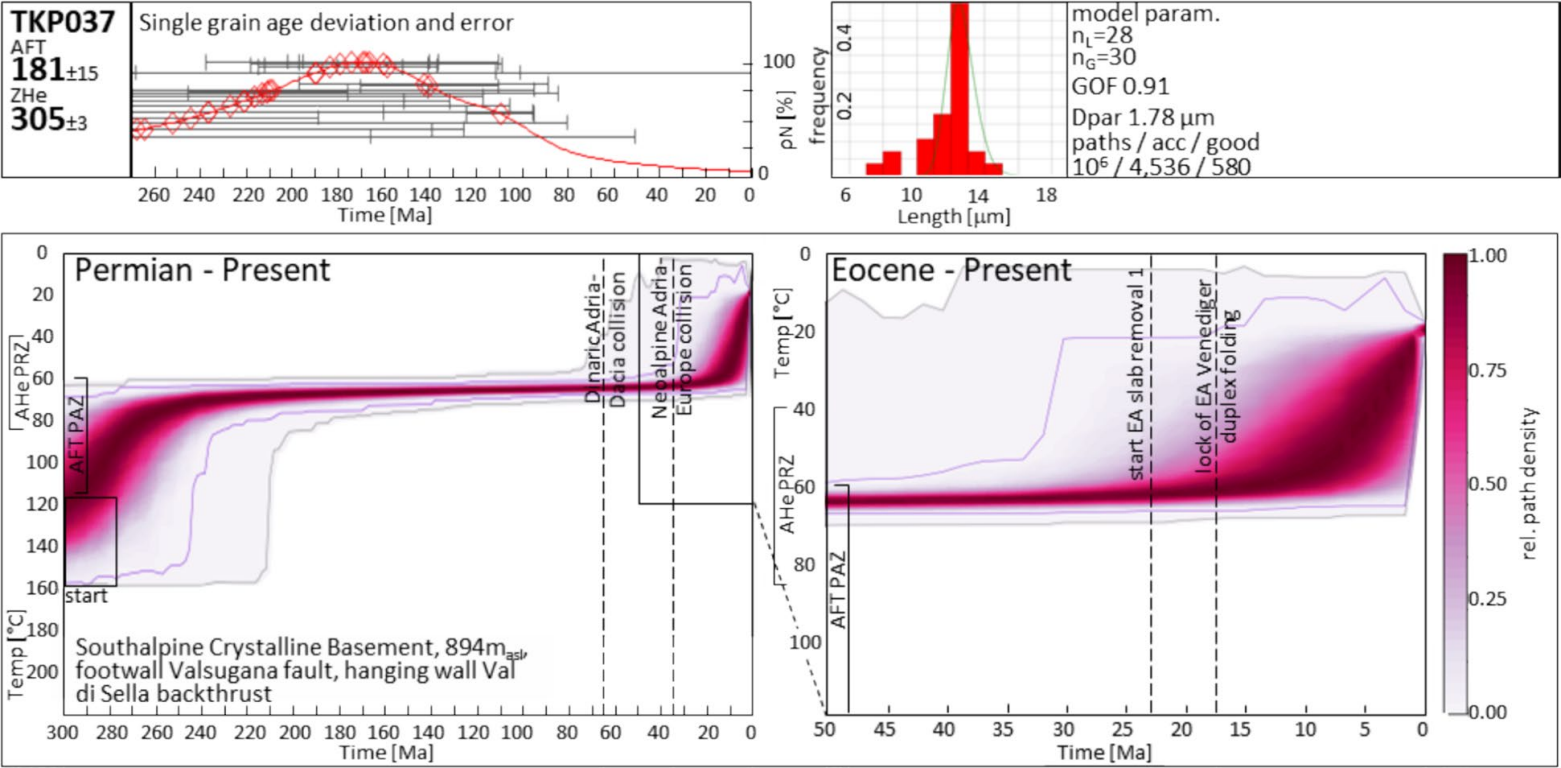
Single-grain ages, track length distribution, and time–temperature path from TKP037, which is located in the hanging wall of the Val di Sella backthrust and, therefore, affected by Bassano-Valdobbiadene fault activity. Starting condition is the AHe date of the sample. The heat map represents a visualized statistic of modelled t-T path occurrences with the purple and grey envelopes limiting the ranges of good and acceptable paths, respectively. *acc* acceptable, *AHe* apatite (U-Th)/He, *AFT* apatite fission-track, Dpar arithmetic mean of AFT etch pit lengths parallel to the crystallographic c-axis (Donelick, 1993), *EA* Eastern Alps, *GOF* goodness of fit, *n*_*L*_ number of measured confined tracks, n_G_ number of analyzed crystals, *PAZ* partial annealing zone, *PRZ* partial retention zone, *rel* relative, *ZHe* zircon (U-Th)/He

Sample TKP037 (Fig. 15, Southalpine Crystalline Basement, hanging wall of the Val di Sella backthrust) exhibits a late Carboniferous ZHe date which suggests that subsequent events did not cause temperatures to increase above ~ 160 °C at the sample location. Its Jurassic AFT apparent age is the oldest in the samples of this study. The associated broad single-grain age distribution in combination with the pronounced track length distribution peak requires a long-lasting Mesozoic residence within the uppermost AFT PAZ (Fig. 15). Pronounced late Neoalpine post-10 Ma cooling in the model clearly attributes uplift at the Val di Sella backthrust to the Adriatic phase and Bassano-Valdobbiadene fault activity. Sample TKP034 (171 ± 26 Ma), from within the Valsugana thrust shear zone, is assumed to originate from an accreted footwall slice due to its similarity to TKP037 (181 ± 15 Ma) in apparent age and single-grain age distribution (Appendix, Sect. 2).

### From cooling to exhumation

**Fig. 16:**
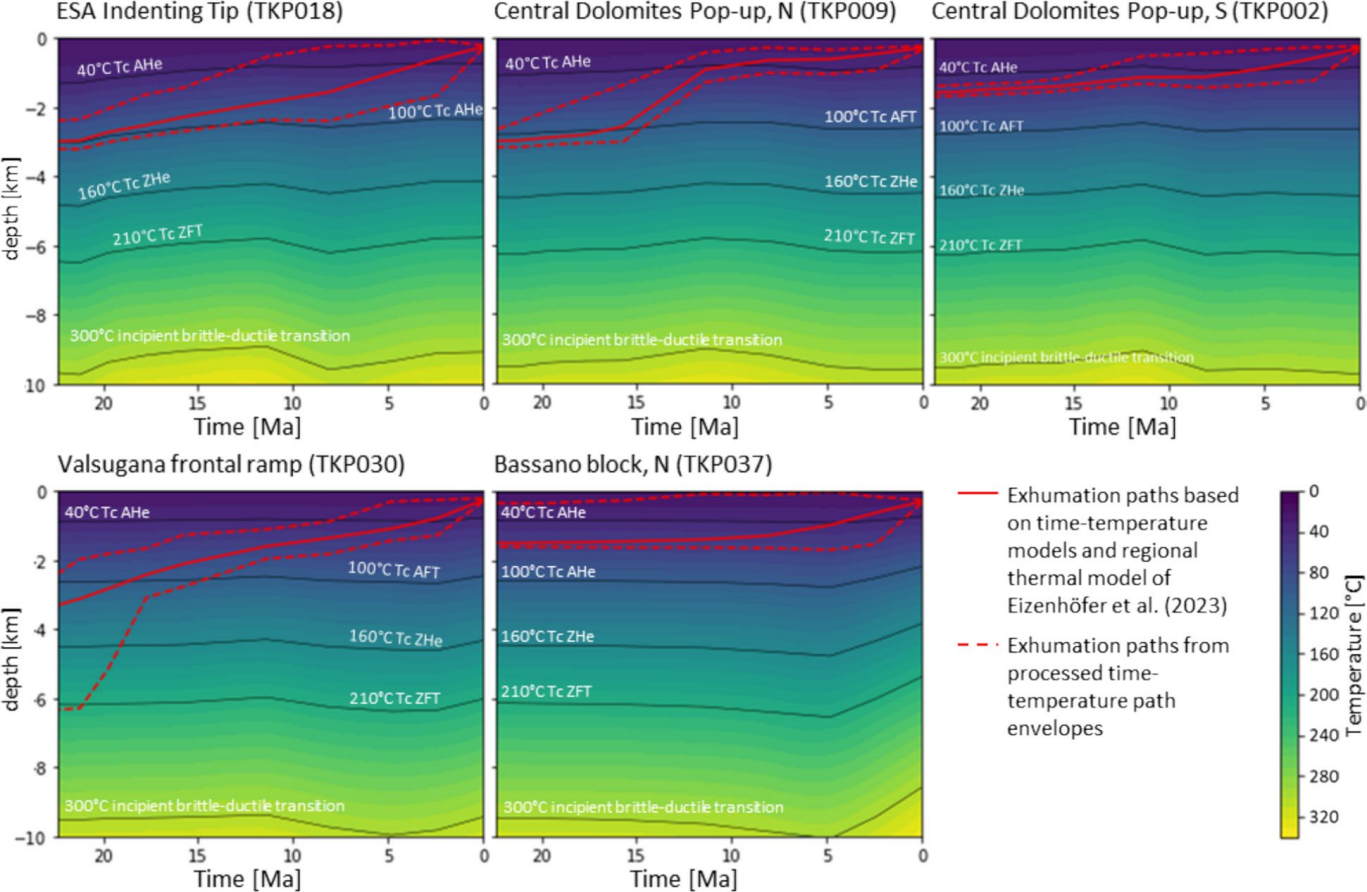
Evolution of the geothermal gradient at the tectonic position of specific samples at the time. Each sample is representative of a tectonic block. The visualization is based on the thermo-kinematic model of Eizenhöfer et al. (2023). Depth represents the modelled overburden at the time

Crustal temperatures and, hence low-temperature thermochronological data, can be affected by transient shifts in the geothermal gradient. Depending on the process, this can occur abruptly (e.g., due to rock motion along active faults) or gradually (e.g., thermal relaxation after a temporary increase in the geothermal gradient), as described by Malusà and Fitzgerald (2019b). Accordingly, the effects of syn- and post-tectonic thermal re-equilibration must be considered when transferring cooling rates to effective exhumation rates. In this study, we employ the thermal model along the TRANSALP geophysical transect by Eizenhöfer et al. (2023). We acknowledge limitations associated with the modelling process as discussed in their publication such as the absence of any lateral offset. Furthermore, the tectonic complexity is simplified to account for the orogen-scale dimensions of their thermal model including a 30 km wide swath to project thermochronological data. Nevertheless, this model is able to trace the thermal history along the TRANSALP geophysical transect back to ~ 22 Ma. Characteristic perturbations of the geothermal field after rock motion and heat transport along active faults, clearly distinguishes the thermal evolution of the samples across the different tectonic blocks along discussed in our study (Fig. 16).

The location of TKP018, representing the ESA Indenting Tip, shows a slight increase of the geothermal gradient in the uppermost 3 km between ~ 21 and ~ 12 Ma, most likely due to piggyback transport on deep-seated ramps or lower crustal thickening. The exhumation path suggests continuous exhumation and a slight acceleration at ~ 8 Ma, which emphasizes the significance of Adriatic/Bassano phase exhumation in the northern ESA. Exhumation rates reach 0.2 km/Myr, which is about half the apparent exhumation rate derived from AFT data of Most (2003).

The exhumation of TKP009 from the northernmost Central Dolomites Pop-Up culminates at a maximum rate of 0.4 km/Myr during the Middle Miocene, probably due to stacked thrusting and backthrusting as discussed in Sect. 6.3.2. This corresponds almost exactly to the apparent exhumation rate derived from the regional age-elevation correlation. Subsequent thermal relaxation during the late Valsugana phase and a slight inclination of the exhumation path is in line with slow piggyback exhumation on nearly flat ramps. TKP002 lacks a strong exhumation pulse, as it was sampled from rock sitting on a flat section of the Valsugana thrust system (Fig. 3). The exhumation rate does not exceed 0.1 km/Myr.

TKP030, representative for the Valsugana thrust hanging wall, suggests a steady exhumation rate of 0.25 km/Myr. However, apparent exhumation rates of 0.5 km/Myr have also been observed. It is noteworthy, that the envelope of good time–temperature paths in our HeFTy models allows for steeper cooling paths. In any case, the exhumation path aligns well to the apparent exhumation derived from AFT data, but appears to undervalue the exhumation rates during the late Valsugana phase.

The exhumation path of TKP037 (Northern Bassano Block) fits well with the expectations of slight Adriatic phase exhumation. A significant tectonic perturbation of the geothermal gradient starts only during Bassano-Valdobbiadene thrust-activity. The post-Miocene maximum of the exhumation rate is 0.15 km/Myr.

## Conclusions

- A new balanced cross section between Mauls and Bassano del Grappa indicates minimum shortening of ~ 18 km. This identifies the central Trento platform as the zone that accommodated the least amount of north–south shortening within the eastern Southern Alps.
- The extensive apatite fission-track dataset contains numerous samples from pre-Mesozoic lithologies but almost no apatite fission-track single-grain ages older than Middle Triassic are preserved. Pre-Mesozoic detrital ages and magmatic formation ages therefore cannot be found throughout the study area. This indicates that temperatures well within the apatite fission-track partial annealing zone were reached.
- Permo-Mesozoic thermal perturbation and sedimentary burial allow for long term residence of the basement top and its lower sedimentary cover within the apatite fission-track partial annealing zone. Fast Neoalpine exhumation preserves these fossil apatite fission-track partial annealing zones.
- Triassic magmatism-triggered heating indicated by time–temperature models on Permian volcanic rocks points out the significant imprint of the Ladinian thermal event.
- The northwest-southeast striking Plose antiform and the southwest-directed St. Ulrich-Klausen blind thrust fault from the Brixen area represent the northwesternmost structures linked to the Eocene Dinaric phase thrusting in the eastern Southern Alps.
- Apatite fission-track and (U-Th)/He data suggest a late Oligocene to Middle Miocene north-side-up tilting of the eastern Southern Alps Indenting Tip.
- The northern Central Dolomites Pop-up exhibits the highest cooling rates within our dataset, taking place between 17 and 10 Ma. This suggests thrusting on a steeply inclined and deep-seated Valsugana ramp and/or coeval backthrusting along the Villnöß and Würzjoch thrusts.
- The southern Central Dolomites Pop-up appears to rest on a flat segment of the Valsugana ramp. Significant cooling is only recorded from the late Tortonian onwards and related to the activity of the Bassano-Valdobbiadene thrust.
- Time–temperature models from the hanging wall of the Valsugana frontal ramp indicate an earlier onset of tectonic activity (~ 25–20 Ma) compared to the central dolomites pop up. This is coeval to the onset of the Giudicarie fault activity, the eastern Southern Alps Indenting Tip north-side-up tilting, and the retro-wedge flexural response.
- Maximum exhumation rates have been calculated based on our new thermal models: The eastern Southern Alps Indenting Tip having experienced ~ 0.2–0.4 km/Myr; the northern Central Dolomites Pop-up ~ 0.3–0.4 km/Myr; the southern Central Dolomites Pop-up < 0.1 km/Myr; the Valsugana frontal ramp 0.25–0.5 km/Myr; and the northern Bassano Block ~ 0.15 km/Myr (affected by thrusting and backthrusting). For the Dinaric exhumation of the Plose massif an apparent exhumation rate (based on its age-elevation relation) of ~ 0.17 km/Myr has been calculated.

## Supplementary Information

Supplementary material 1.

## Data Availability

The datasets generated during the current study are available in the GFZ Data Services repository, 10.5880/fidgeo.2025.018 (Klotz et al., 2025). Code generated is available in the GFZ Data Services repository 10.5880/fidgeo.2025.019 (Klotz, 2025).

## Abbreviations

AFT: Apatite fission-track
AHe: Apatite (U-Th)/He
ESA: Eastern Southern Alps
GFS: Giudicarie fault system
ZHe: Zircon (U-Th)/He
