# Supplementary material for: A thermochronological transect across the Trento platform: constraints for the evolution of the European Eastern Southern Alps
Source: Swiss J Geosci. 2025 Sep 25;118(1):18. doi: 10.1186/s00015-025-00491-w (PMC12464088; doi:10.1186/s00015-025-00491-w)
Supplement: Supplementary file 1 — Supplementary material 1. [file 15_2025_491_MOESM1_ESM.docx]

A Thermochronological Transect across the Trento Platform – Constraints for the Evolution of the European Eastern Southern Alps - APPENDIX

Thomas Klotz^1^, Anna-Katharina Sieberer^1^, István Dunkl^2^, Paul R. Eizenhöfer^3^, Hannah Pomella^1^

^1^University of Innsbruck, Department of Geology, Innsbruck, 6020, Austria

^2^University of Göttingen, Department of Sedimentology and Environmental Geology, Göttingen, 37077, Germany

^3^University of Glasgow, School of Geographical & Earth Sciences, Glasgow, G12 8QQ, Scotland

Corresponding author: Thomas Klotz ([thomas.klotz@uibk.ac.at)](mailto:thomas.klotz@uibk.ac.at))

Co-Autors: Anna-Katharina Sieberer ([anna-katharina.sieberer@uibk.ac.at](mailto:anna-katharina.sieberer@uibk.ac.at)), István Dunkl ([istvan.dunkl@geo.uni-goettingen.de](mailto:istvan.dunkl@geo.uni-goettingen.de)), Paul R. Eizenhöfer ([paul.eizenhoefer@glasgow.ac.uk](mailto:paul.eizenhoefer@glasgow.ac.uk)), Hannah Pomella ([hannah.pomella@uibk.ac.at](mailto:hannah.pomella@uibk.ac.at))

1. **Methods**

Apatite fission-track (AFT) analysis is a statistical and geometric application based on the spontaneous fission of ^238^U. Lattice damage, induced by diametral extraction of fission particles, forms straight fission-tracks with an initial length of 21.2±0.9 µm, of which ~16 µm are etchable for microscopic examination (Jonckheere, 2003; Ketcham, 2019). Fission-tracks anneal instantaneously at high temperatures but are generated within and below the partial annealing zone (PAZ), which is in the range of 115-60°C (Carlson et al., 1999; Gallagher et al., 1998; Green et al., 1986; Malusà & Fitzgerald, 2019). Residence within the PAZ shortens fission-tracks. Shortening is a function of cooling rate and temperature. Statistical analyses of track lengths can be used to trace the thermal history (Ketcham, 2019, and references therein). We used the external detector method (Gleadow, 1981) and samples were exposed to a nominal dose of 4.5 x 10^15^ n cm^-2^ of neutron irradiation at the Oregon State University Radiation Center research reactor. For the quantification of irradiation intensity IRMM540 dosimeter glass were used. Apatites were etched in 5.5 M HNO_3_ at 20°C for 20 s, induced tracks on the external muscovite detector in 40% HF for 45 min at 20°C following the standard protocol of Ketcham et al. (2015). Age data were calculated using the Trackkey software version 4.2g (Dunkl, 2002). For age calculation we used the zeta calibration method (Hurford & Green, 1983) with a zeta of 250.42 ± 14.79, based on Fish Canyon Tuff (27.9 ± 0.5 Ma, McDowell & Kreizer, 1977) and Durango apatite (31.4 ± 0.5 Ma, Hurford & Hammerschmidt, 1985) apatite. The sample processing procedures for zircon fission-track (ZFT) are similar to those of the AFT method. Since we are using only compiled ZFT data, we refer descriptions by Hurford and Green (1983), who specify a temperature range of 180-300°C as the ZFT PAZ.

Single grain apatite (U-Th)/He (AHe) analysis considers the retention of He-atoms which results from the α-decay of ^238^U, ^235^U, ^232^Th, and ^147^Sm within the crystal lattice (Wolf et al., 1996). He-retention in apatite occurs at temperatures below 85°C, with a partial retention zone (PRZ) above 40°C, though the extend of this PRZ is dependent on various factors including the bulk rock cooling rate, grain radiation damage, grain dimensions and tectonic strain (Farley, 2002; Wolf et al., 1998). Farley (2000) proposes a closure temperature of ~68°C for the AHe system, which should prove useful for the majority of geologic settings. Apatite crystals were selected only when free of crystalline or fluid inclusions, and preferably when euhedral and intact. The grain size was restricted to a minimum of 60 µm with respect to the smallest dimension. Deviations in color and the presence of obvious zoning were criteria used to potentially exclude specific crystals. The closure temperature for single grain zircon (U-Th)/He (ZHe) analysis is not well constrained. Reiners et al. (2004) obtained closure temperatures of 171-196°C, while a more recent publication by Yu et al. (2019) suggests 136°C for slow cooling and 199°C for rapid cooling, with an average of 176°C. He-extraction and measurements, as well as U, Th, and Sm measurement, were conducted in the low-T geochronology lab of the Geoscience Center at the University of Göttingen.

To retrace the thermal history of the samples, an inversion of the AFT and AHe data was performed with the HeFTy software v2.1.7 (Ketcham, 2005, 2024; Ketcham et al., 2007). The merit value thresholds were set at 0.5 for “acceptable” time-temperature paths and 0.05 for “good” time-temperature paths. Additional statistical constraints were obtained by visualizing a graphical mean contour map, which was generated using exported time-temperature paths. We used the following general HefTy inverse modelling parameters: Path between constraints “Monotonic variable”; Halve 4 times; Randomizer style “Episodic”; no dT/dt restrictions; Search method “Monte Carlo”; The following general HefTy AFT model parameters were applied: Annealing model Ketcham et al. (2007); C-axis projection Ketcham et al. (2007), 5.5M; AHe model parameters: Calibrations Farley (2000, Durango Apatite); Stopping distances Ketcham et al. (2011); Alpha calculation “redistribution”

1. **Supporting Data**

The figures in this section display the AHe data (Fig. AP2.1) and the AFT data (Figs. AP2.2 – AP2.6) in an additional type of presentation to provide statistical information and allow for enhanced data validation. Parameters referring to modelling, concentration calculations, and the quality of the measurements are given in Table AP2.1 for apatite (U-Th-Sm)/He data, and in Table AP2.2 for zircon (U-Th-Sm)/He data.


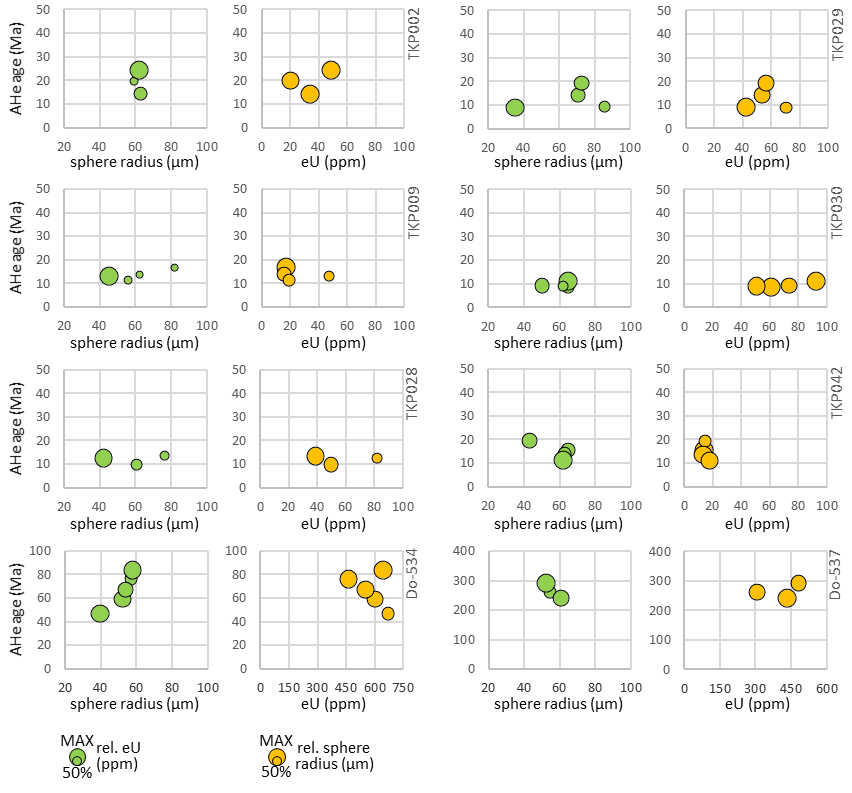


Fig. AP2.1: AHe eU vs. age and equivalent spherical radius vs. age, based on suggestions of (Flowers, Ketcham, et al., 2022; Flowers, Zeitler, et al., 2022).


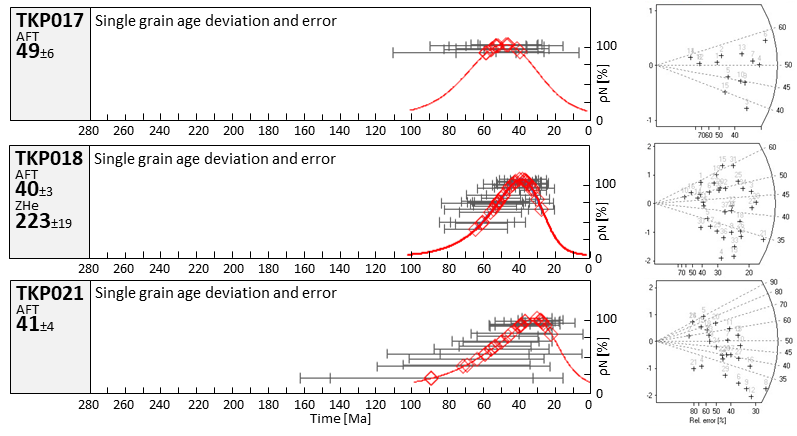


Fig. AP2.2: Single-grain age plots and radial plots of AFT data from the ESA Indenting Tip


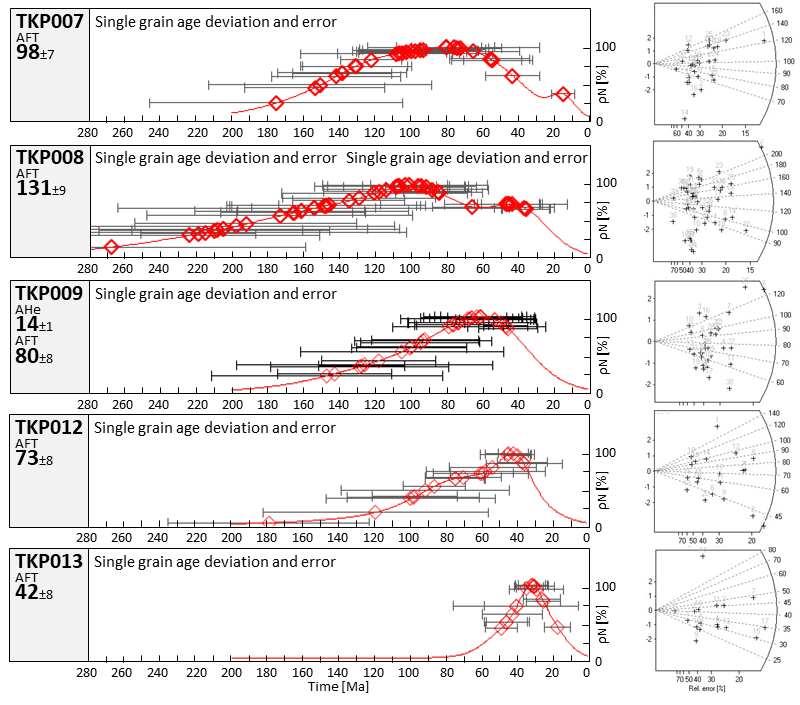


Fig. AP2.3: Single-grain age plots and radial plots of AFT data from the northern Central Dolomites Pop-up (Schlern region and Val Gardena)


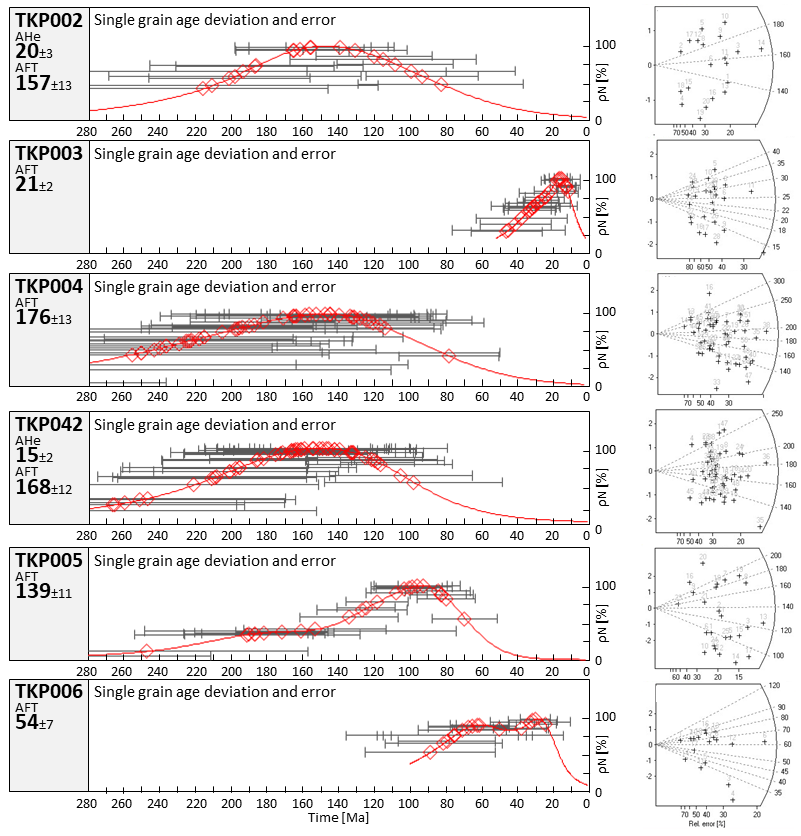


Fig. AP2.4: Single-grain age plots and radial plots of AFT data from the southern Central Dolomites Pop-up (Predazzo, Val d’Adige)


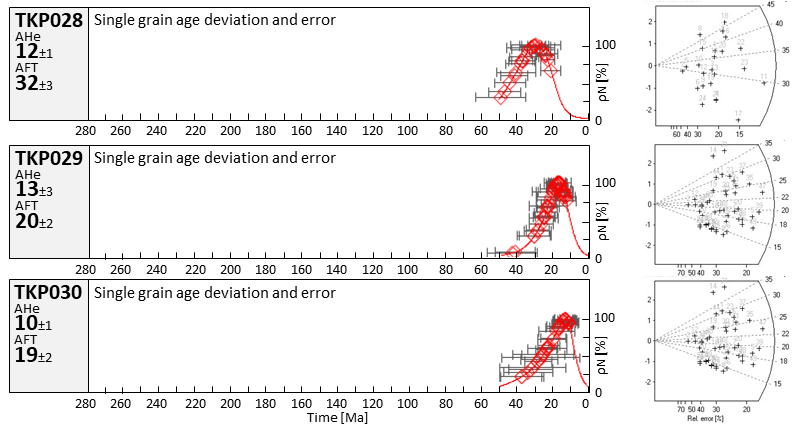


Fig. AP2.5: Single-grain age plots and radial plots of AFT data from the Valsugana Frontal Ramp


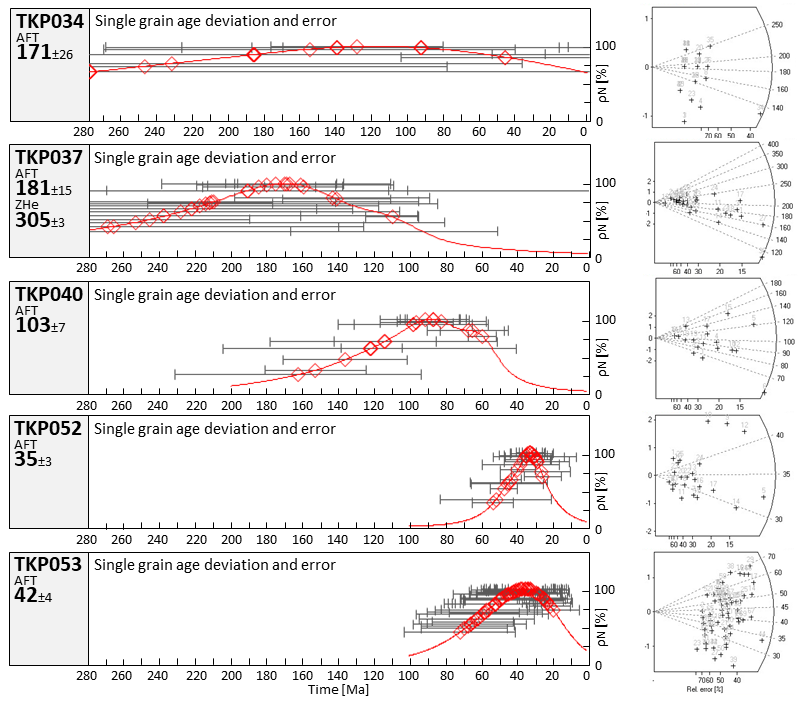


Fig. AP2.6: Single-grain age plots and radial plots of AFT data from the Valsugana Frontal Ramp

| Table AP2.1: Apatite (U-Th-Sm)/He data; calculations and quality | | | | | | | | | | |
| --- | --- | --- | --- | --- | --- | --- | --- | --- | --- | --- |
| **Sample** | grain | modelling and concentration calculations | | | |  |  | Quality of the measurement | | |
|  | ID | sphere radius [µm] | crystal mass by size [µg] | crystal mass by ICP [µg] | eU [ppm] | Rad. density [He nmol/g] |  | TAU [%] | Estim. error of Ft [%] | First He re-extract [%] |
| **TKP002** | a1 | 59 | 4.6 | 4.5 | 20.5 | 1.9 |  | 2.1 | 4.1 | 1.3 |
|  | a2 | 63 | 2.4 | 2.2 | 33.8 | 2.3 |  | 2.3 | 3.8 | 2.4 |
|  | a3 | 62 | 2.5 | 2.5 | 48.4 | 5.4 |  | 2.1 | 3.8 | 1.6 |
| **TKP009** | a1 | 82 | 8.4 | 7.5 | 17.2 | 1.4 |  | 2.1 | 2.9 | 0.9 |
|  | a2 | 62 | 4.5 | 4.0 | 15.6 | 1.1 |  | 2.2 | 3.8 | 1.2 |
|  | a3 | 56 | 4.1 | 4.2 | 19.1 | 1.0 |  | 2.2 | 4.5 | 0.8 |
|  | a4 | 45 | 1.6 | 1.5 | 47.0 | 2.3 |  | 2.5 | 5.6 | 0.6 |
| **TKP028** | a1 | 60 | 2.5 | 2.1 | 49.7 | 2.2 |  | 2.1 | 3.8 | 4.8 |
|  | a2 | 76 | 4.7 | 4.6 | 38.8 | 2.6 |  | 2.0 | 2.8 | 4.5 |
|  | a3 | 42 | 1.8 | 1.7 | 81.8 | 3.8 |  | 2.2 | 5.5 | 6.3 |
| **TKP029** | a1 | 86 | 11.9 | 11.5 | 42.5 | 1.9 |  | 2.0 | 2.7 | 1.7 |
|  | a2 | 35 | 1.1 | 1.2 | 70.8 | 2.0 |  | 2.5 | 6.7 | 0.7 |
|  | a3 | 70 | 5.4 | 5.4 | 53.8 | 3.5 |  | 2.1 | 3.3 | 2.0 |
|  | a4 | 73 | 7.0 | 6.8 | 56.6 | 5.0 |  | 1.9 | 3.3 | 0.5 |
| **TKP030** | a1 | 65 | 4.2 | 4.1 | 60.7 | 2.3 |  | 2.1 | 3.6 | 5.8 |
|  | a2 | 50 | 2.7 | 2.6 | 73.5 | 2.7 |  | 2.1 | 4.7 | 0.4 |
|  | a3 | 65 | 4.5 | 4.2 | 92.7 | 4.5 |  | 2.1 | 3.6 | 2.0 |
|  | a4 | 62 | 3.4 | 3.2 | 50.6 | 2.0 |  | 2.2 | 3.9 | 0.6 |
| **TKP042** | a1 | 65 | 3.7 | 4.0 | 13.6 | 1.1 |  | 2.2 | 3.6 | 0.4 |
|  | a2 | 43 | 2.0 | 2.1 | 14.5 | 1.0 |  | 3.1 | 5.7 | 0.8 |
|  | a3 | 63 | 2.9 | 2.7 | 13.2 | 0.8 |  | 2.7 | 3.8 | 0.6 |
|  | a4 | 62 | 3.2 | 2.9 | 17.7 | 0.9 |  | 2.5 | 3.8 | 4.6 |
| **Do-534** | a1 | 41 | 2.0 | 2.2 | 46.3 | 1.1 |  | 3.0 | 5.8 | 0.3 |
|  | a2 | 40 | 0.9 | 0.9 | 64.0 | 1.5 |  | 3.6 | 5.9 | 0.9 |
|  | a3 | 41 | 1.2 | 1.2 | 14.8 | 0.6 |  | 5.5 | 5.8 | 2.0 |
|  | a4 | 48 | 1.8 | 1.7 | 42.6 | 1.2 |  | 3.3 | 5.0 | 0.4 |
| **Do-537** | a1 | 31 | 0.7 | 1.1 | 19.8 | 0.4 |  | 6.1 | 7.8 | 3.8 |
|  | a2 | 62 | 3.6 | 4.0 | 27.5 | 1.3 |  | 2.3 | 3.9 | 1.8 |
|  | a3 | 35 | 1.0 | 1.1 | 25.2 | 0.5 |  | 5.9 | 6.9 | 1.3 |
|  | a4 | 41 | 1.5 | 2.1 | 17.2 | 0.8 |  | 3.6 | 6.1 | 1.7 |

| Table AP2.2: Zircon (U-Th-Sm)/He data; calculations and quality | | | | | | | | | | |
| --- | --- | --- | --- | --- | --- | --- | --- | --- | --- | --- |
| **Sample** | grain | modelling and concentration calculations | | | |  |  | Quality of the measurement | | |
|  | ID | sphere radius [µm] | crystal mass by size [µg] | crystal mass by ICP [µg] | eU [ppm] | Rad. density [He nmol/g] |  | TAU [%] | Estim. error of Ft [%] | First He re-extract [%] |
| **TKP018** | a1 | 54 | 3.3 | 4.2 | 176.8 | 124.9 |  | 2.2 | 3.5 | 6.1 |
|  | a2 | 55 | 5.4 | 5.2 | 117.6 | 110.6 |  | 2.2 | 3.3 | 6.8 |
|  | a3 | 49 | 3.9 | 3.7 | 246.5 | 251.0 |  | 2.2 | 3.7 | 27.0 |
|  | a4 | 49 | 2.8 | 2.8 | 289.8 | 300.6 |  | 2.1 | 3.7 | 9.8 |
| **TKP037** | a1 | 54 | 4.5 | 6.0 | 375.9 | 473.9 |  | 2.2 | 3.5 | 29.9 |
|  | a2 | 61 | 4.9 | 5.9 | 413.5 | 557.3 |  | 2.2 | 3.1 | 27.3 |
|  | a3 | 77 | 8.9 | 10.6 | 230.0 | 319.9 |  | 2.2 | 2.5 | 7.2 |
| **6-47** | a1 | 55 | 7.7 | - | 851.4 | 613.5 |  | 2.3 | 3.4 | 5.6 |
|  | a2 | 63 | 11.7 | - | 741.8 | 415.8 |  | 2.3 | 3.0 | 4.2 |
|  | a3 | 68 | 10.4 | - | 651.9 | 512.4 |  | 2.3 | 2.8 | 1.0 |
| **9-17** | a1 | 54 | 8.3 | - | 308.1 | 339.9 |  | 2.3 | 3.4 | 1.1 |
|  | a2 | 61 | 9.0 | - | 431.5 | 453.1 |  | 2.3 | 3.1 | 3.1 |
|  | a3 | 52 | 6.2 | - | 480.6 | 589.5 |  | 2.3 | 3.5 | 6.7 |
| **Do-502** | a1 | 42 | 2.2 | 1.9 | 650.7 | 528.1 |  | 1.9 | 4.3 | 0.7 |
|  | a2 | 50 | 3.9 | 2.9 | 738.3 | 346.6 |  | 1.9 | 3.7 | 7.1 |
|  | a3 | 53 | 4.8 | 5.1 | 143.3 | 121.7 |  | 1.9 | 3.5 | 1.5 |
|  | a4 | 38 | 1.7 | 1.6 | 758.1 | 505.2 |  | 1.9 | 4.7 | 10.2 |
|  | a5 | 45 | 3.4 | 3.4 | 586.5 | 474.8 |  | 1.9 | 4.1 | 11.9 |

1. **Discussion – Additional Information**

**
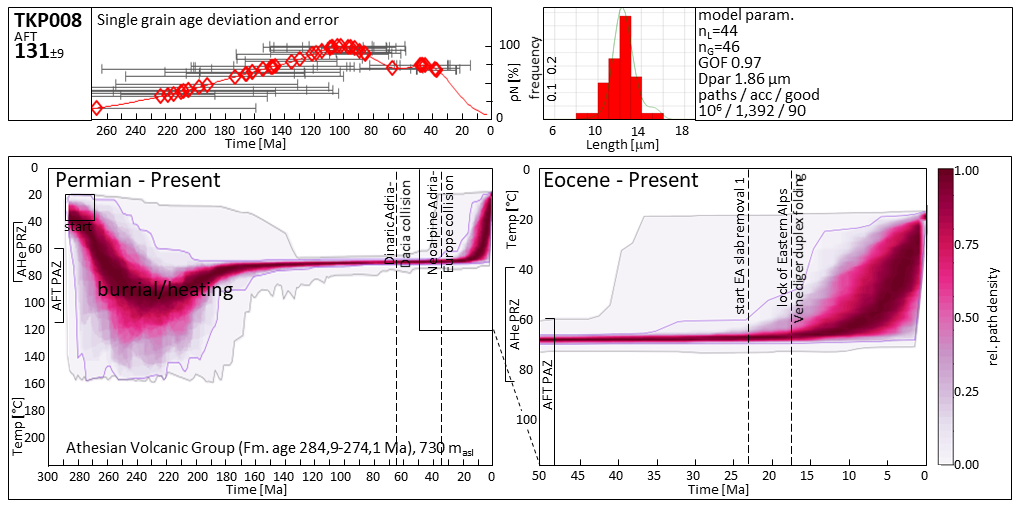
**

Fig. AP3.1: Single-grain age, track length distribution, and time-temperature paths from TKP008 in the Central Dolomites Pop-up. chi^2^ P = 0,0%. Starting condition is the respective deposition age of the sedimentary rocks. Therefore, subsequent burial or heating is required. The heat map represents a visualized statistic of modelled t-T path occurrences with the purple and grey envelopes limiting the ranges of good and acceptable paths, respectively.

**References**

Carlson, W. D., Donelick, R. A., & Ketcham, R. A. (1999). Variability of apatite fission-track annealing kinetics: I. Experimental results. *American Mineralogist*, *84*(9), 1213-1223.

Dunkl, I. (2002). Trackkey: a Windows program for calculation and graphical presentation of fission track data. *Computers & Geosciences*, *28*(1), 3-12.

Farley, K. A. (2000). Helium diffusion from apatite: General behavior as illustrated by Durango fluorapatite. *Journal of Geophysical Research: Solid Earth*, *105*(B2), 2903-2914. <https://doi.org/https://doi.org/10.1029/1999JB900348>

Farley, K. A. (2002). (U-Th)/He Dating: Techniques, Calibrations, and Applications. *Reviews in Mineralogy and Geochemistry*, *47*(1), 819-844. <https://doi.org/10.2138/rmg.2002.47.18>

Flowers, R. M., Ketcham, R., Enkelmann, E., Gautheron, C., Reiners, P. W., Metcalf, J. R., Danišík, M., Stockli, D., & Brown, R. W. (2022). (U-Th)/He chronology: Part 2. Considerations for evaluating, integrating, and interpreting conventional individual aliquot data. *GSA Bulletin*, *135*. <https://doi.org/10.1130/B36268.1>

Flowers, R. M., Zeitler, P. K., Danišík, M., Reiners, P. W., Gautheron, C., Ketcham, R., Metcalf, J. R., Stockli, D., Enkelmann, E., & Brown, R. W. (2022). (U-Th)/He chronology: Part 1. Data, uncertainty, and reporting. *GSA Bulletin*, *135*, 104-136. <https://doi.org/10.1130/B36266.1>

Gallagher, K., Brown, R., & Johnson, C. (1998). Fission track analysis and its application to geological problems. *Annual Review of Earth and Planetary Sciences*, *26*, 519-572. <https://doi.org/doi:10.1146/annurev.earth.26.1.519>

Gleadow, A. J. W. (1981). Fission-track dating methods: what are the real alternatives? *Nuclear Tracks*, *5*, 3-14.

Green, P. F., Duddy, I. R., Gleadow, A. J. W., Tingate, P. R., & Laslett, G. M. (1986). Thermal annealing of fission tracks in apatite: 1. A qualitative description. *Chemical Geology*, *59*(4), 237-253. <https://doi.org/https://doi.org/10.1016/0168-9622(86)90074-6>

Hurford, A. J., & Green, I. R. (1983). The zeta age calibration of fission track dating. *Isotope Geoscience*, *1*, 285-317.

Hurford, A. J., & Hammerschmidt, K. (1985). 40Ar/39Ar and K/Ar dating of the Bishop an Fish Canyon Tuffs: calibration ages for fission track dating standards. *Chemical Geology: Isotope Geoscienze section*, *58*(1-2), 23-32.

Jonckheere, R. (2003). On the densities of etchable fission tracks in a mineral and co-irradiated external detector with reference to fission-track dating of minerals. *Chemical Geology*, *200*, 41-58. <https://doi.org/10.1016/S0009-2541(03)00116-5>

Ketcham, R. (2019). Fission-Track Annealing: From Geologic Observations to Thermal History Modeling. In M. G. Malusà & P. G. Fitzgerald (Eds.), *Fission-Track Thermochronology and its Application to Geology* (pp. 49-75). Springer International Publishing. <https://doi.org/10.1007/978-3-319-89421-8_3>

Ketcham, R. A. (2005). Forward and Inverse Modeling of Low-Temperature Thermochronometry Data. *Reviews in Mineralogy and Geochemistry*, *58*(1), 275-314. <https://doi.org/10.2138/rmg.2005.58.11>

Ketcham, R. A. (2024). Thermal history inversion from thermochronometric data and complementary information: New methods and recommended practices. *Chemical Geology*, *653*, 122042. <https://doi.org/https://doi.org/10.1016/j.chemgeo.2024.122042>

Ketcham, R. A., Carter, A., Donelick, R. A., Barbarand, J., & Hurford, A. J. (2007). Improved modeling of fission-track annealing in apatite. *American Mineralogist*, *92*(5-6), 799-810.

Ketcham, R. A., Carter, A., & Hurford, A. J. (2015). Inter-laboratory comparison of fission track confined length and etch figure measurements in apatite. *100*(7), 1452-1468. <https://doi.org/doi:10.2138/am-2015-5167> (American Mineralogist)

Malusà, M. G., & Fitzgerald, P. G. (2019). Application of Thermochronology to Geologic Problems: Bedrock and Detrital Approaches. In M. G. Malusà & P. G. Fitzgerald (Eds.), *Fission-Track Thermochronology and its Application to Geology* (pp. 191-209). Springer International Publishing. <https://doi.org/10.1007/978-3-319-89421-8_10>

McDowell, F. W., & Kreizer, P. (1977). Timing of mid-Tertiary volcanism in the Sierra Madre Occidental between Durango City and Mazatlan, Mexico. *Geological Society of America Bulletin*, *88*(10), 1479-1487.

Reiners, P. W., Spell, T. L., Nicolescu, S., & Zanetti, K. A. (2004). Zircon (U-Th)/He thermochronometry: He diffusion and comparisons with 40Ar/39Ar dating. *Geochimica et Cosmochimica Acta*, *68*(8), 1857-1887. <https://doi.org/https://doi.org/10.1016/j.gca.2003.10.021>

Wolf, R., Farley, K., & Silver, L. (1996). Helium diffusion and low-temperature thermochronometry of apatite. *Geochimica et Cosmochimica Acta*, *60*(21), 4231-4240.

Wolf, R. A., Farley, K. A., & Kass, D. M. (1998). Modeling of the temperature sensitivity of the apatite (U–Th)/He thermochronometer. *Chemical Geology*, *148*(1), 105-114. <https://doi.org/https://doi.org/10.1016/S0009-2541(98)00024-2>

Yu, S., Chen, W., Sun, J., & Shen, Z. (2019). Diffusion of helium in FCT zircon. *Science China Earth Sciences*, *62*(4), 719-732. <https://doi.org/10.1007/s11430-017-9283-3>
